# Factors Influencing the Colostrum’s Microbiota: A Systematic Review of the Literature

**DOI:** 10.3390/children12101336

**Published:** 2025-10-04

**Authors:** Aimilia Tzani, Nikoleta Aikaterini Xixi, Rozeta Sokou, Eleni Karapati, Zoi Iliodromiti, Paraskevi Volaki, Styliani Paliatsiou, Nikoletta Iacovidou, Theodora Boutsikou

**Affiliations:** 1 School of Medicine, National and Kapodistrian University of Athens, 11528 Athens, Greece; aimilia.tzani@hug.ch; 2 Neonatal Department, Aretaieio Hospital, School of Medicine, National and Kapodistrian University of Athens, 11528 Athens, Greece; nerinaxixi@med.uoa.gr (N.A.X.); helenakar@med.uoa.gr (E.K.); ziliodromiti@med.uoa.gr (Z.I.); voulavolaki@med.uoa.gr (P.V.); stpaliatsiou@med.uoa.gr (S.P.); niakobid@med.uoa.gr (N.I.); theobtsk@med.uoa.gr (T.B.)

**Keywords:** breastfeeding, microbiome, gut colonization, bacteria, probiotics

## Abstract

**Background/Objectives:** Human colostrum plays a crucial role in early microbial colonization, immune development, and gut health of newborns. Its microbiota is highly dynamic and influenced by numerous factors, yet the determinants remain poorly understood. This systematic review aims to investigate the composition of colostrum microbiota and the intrinsic and extrinsic factors that influence its diversity and abundance. **Methods:** PubMed and Scopus were systematically searched using a prespecified search phrase. Data on microbial composition, diversity, and influencing factors were extracted and analyzed. The systematic review is registered in PROSPERO (CRD42025644017). **Results:** A total of 44 eligible studies involving 1982 colostrum samples were identified. Colostrum microbiota consists predominantly of *Firmicutes* and *Proteobacteria*, with core genera including *Staphylococcus*, *Streptococcus*, *Lactobacillus*, and *Bifidobacterium*. Some studies reported higher diversity in colostrum compared to mature milk, while others noted elevated bacterial abundance in the former. Factors influencing colostrum microbiota include maternal BMI, delivery mode, gestational age, diet, gestational diabetes mellitus (GDM), maternal stress, maternal age, secretor status, perinatal antibiotic exposure, neonatal gender, geographic location, feeding type, milk collection method, and mastitis. **Conclusions:** Colostrum hosts a diverse and dynamic microbiota shaped by multiple maternal, neonatal, and environmental factors. Understanding these influences is crucial for optimizing infant health outcomes, emphasizing the need for further research on the functional roles of colostrum’s microbiota.

## 1. Introduction

Human milk is widely recognized as the optimal source of nutrition for infants, providing a dynamic blend of nutritional and bioactive components tailored to meet the needs of newborns. Its composition is not static, and it changes by various factors such as the stage of lactation, maternal health, and environmental influences [1,2,3]. Human colostrum is uniquely adapted to satisfy the early immunological and nutritional requirements of neonates. Compared to mature milk, colostrum contains higher concentrations of immunoglobulins, growth factors, cytokines, immune cells, and other bioactive compounds that confer immune protection, support gut health, and offer immediate defense against infections [1,2,3,4,5,6].

Breastfeeding offers a well-known range of significant short- and long-term health benefits for infants. It protects against infections, reduces the risk of necrotizing enterocolitis, and minimizes allergic conditions [7,8,9,10,11]. Breastfed infants may also have a lower likelihood of obesity later in life and reduced risk of metabolic syndrome in adulthood. In addition, studies suggest that breastfed children often perform better on intelligence tests, suggesting cognitive benefits associated with breastfeeding [12,13].

Colostrum, provided through early breastfeeding, provides even more distinct and immediate health benefits, particularly for populations such as preterm or low-birth-weight infants that face many challenges postpartum. Early breastfeeding reduces neonatal mortality, lowers the incidence of necrotizing enterocolitis, offers protection against late-onset sepsis, leads to faster achievement of full enteral feeding, and quicker recovery to birth weight, parameters that are crucial for the survival and health optimization of susceptible infants [14,15,16].

Growing research data suggests that breast milk hosts its own unique microbiota, which may play a crucial role in promoting health benefits both for mothers by supporting mammary gland health and infants by aiding gut colonization, thus providing defense against pathogens, fostering immune system development, and assisting in nutrient digestion [17].

The initial microbial colonization of infants is accelerated rapidly after birth, primarily derived from the maternal microbiota, which serves as the infant’s main microbial source [18]. Maternal breast milk, especially colostrum, plays a significant role in this process, as it introduces beneficial microbes that contribute to infant gut colonization and supports infant but also maternal health [19]. Differences in gut microbiota between breastfed and formula-fed infants underscore the importance of this microbial transfer. Studies show that exclusively breastfed infants exhibit gut microbiota with lower diversity, yet higher relative abundance of beneficial bacteria, when compared to formula-fed infants [20,21].

The composition of an infant’s intestinal microbiota is essential for immune system development and is linked to various long-term health outcomes. A disrupted or imbalanced microbiota in infancy is associated with increased risk of allergic conditions, chronic inflammatory bowel disease, obesity, type 1 Diabetes, and reduced vaccine efficacy [20,22,23,24,25,26].

Despite the recognized role of breast milk microbiota in early-life health, limited information exists on the composition and variability of microbiota, especially for colostrum, and even less is understood about the factors influencing it. This systematic review aims to summarize current evidence on the microbiota of human colostrum, examining its composition, diversity, and potential roles in neonatal gut colonization and immune development. It also seeks to identify intrinsic (e.g., maternal health, mode of delivery) and extrinsic (e.g., environment, diet, antibiotics) factors that influence colostrum microbiota, and to highlight gaps in knowledge to guide future research on early-life microbial transfer.

## 2. Materials and Methods

This systematic review is in accordance with the Preferred Reporting Items for Systematic Reviews and Meta-Analyses (PRISMA) statement [27]. A prespecified protocol was formulated and registered in PROSPERO (CRD42025644017) and is available online. Full protocol details can be found in the Data Supplement.

### 2.1. Search Strategy

Two authors (AT and NAX) independently conducted the literature search. Any discrepancy was solved by discussion between them. PubMed and Scopus were systematically searched along with the references of retrieved articles. A search phrase was formulated using Boolean logic and a combination of terms related to human milk and its microbial content. The main keywords included the following: “human milk,” “breast milk,” “colostrum,” “microbiota,” “microbiome,” “microflora,” and “flora”. “Human milk,” “breast milk,” and “colostrum” represent different stages and terminologies of lactation, while “microbiota,” “microbiome,” “microflora,” and “flora” encompass various terms used in the literature to describe microbial communities. All relevant literature in English up until 17 June 2025 was retrieved with no time restrictions. We considered for inclusion studies providing collective data on colostrum microbiota, which was defined as the milk produced during the first 5 days postpartum. Studies were excluded if they (i) studied pasteurized milk; (ii) did not clearly specify the type of milk analyzed; or (iii) involved milk collected after five days postpartum. Additionally, reference lists of selected articles were reviewed to identify other potentially relevant studies. All observational studies (cohort studies, cross-sectional studies, case–control studies, case series, case reports) and clinical trials referring to colostrum’s microbiota will be included. Review articles, systematic reviews, and meta-analyses, as well as conference proceedings, will be excluded.

### 2.2. Data Extraction

A prespecified table including data on the name of the first author, publication year, country of research, participant characteristics (e.g., maternal age, prior antibiotic use, probiotic supplementation, gestational age, mode of delivery, feeding method), milk sample size, timing and method of milk collection, microbiota analysis techniques, and key findings related to microbial diversity, abundance, and associations with influencing factors was utilized. Data extraction and quality assessment were performed independently by two authors (AT, EK). Any disagreements between the initial reviewers were resolved through discussion; if a consensus could not be reached, a third reviewer was involved to achieve resolution (NAX).

### 2.3. Outcomes

The primary outcomes of interest were the following: (1) the identification and characterization of colostrum microbiota, with particular attention to dominant genera and species reported across studies, and (2) the factors influencing colostrum colonization, including maternal characteristics (e.g., health status, antibiotic use), mode of delivery, gestational age, and environmental or hospital-related exposures.

## 3. Results

**Figure 1:**
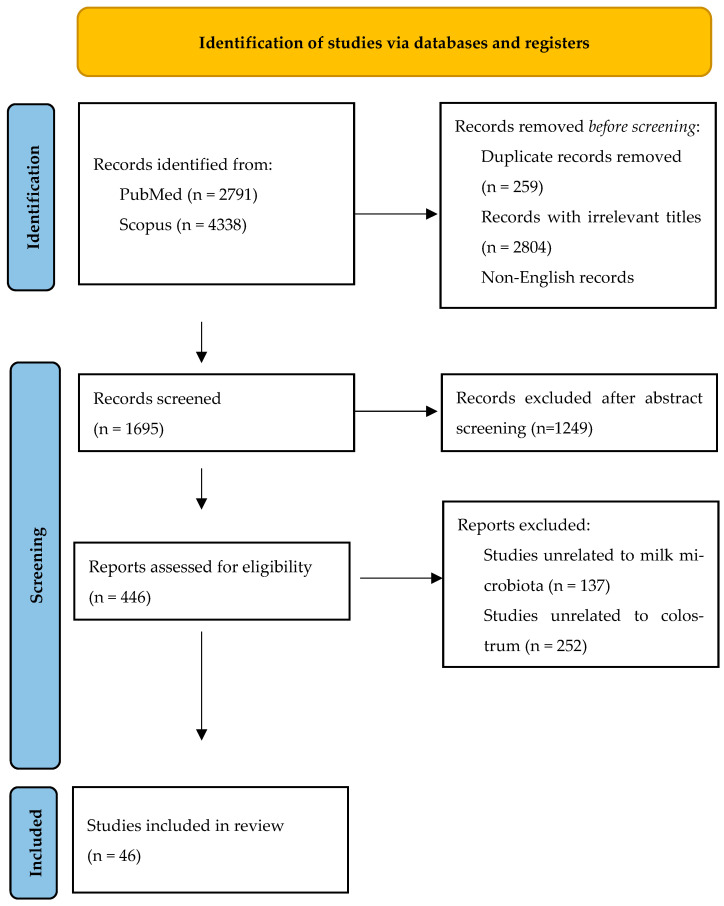
PRISMA flow diagram.

A total of 7129 articles were initially retrieved, 2791 of which were from PubMed and 4338 from Scopus. After eliminating 2597 duplicates, 2804 records with irrelevant titles, and 33 non-English records, 1698 articles remained for abstract screening. Of these, 446 studies were sought for retrieval, and 41 studies were included in the final analysis. Additionally, 5 relevant studies were identified through manual reference searching, bringing the total number of studies included in this review to 46, as depicted in the PRISMA flow diagram (Figure 1).

**Figure 2:**
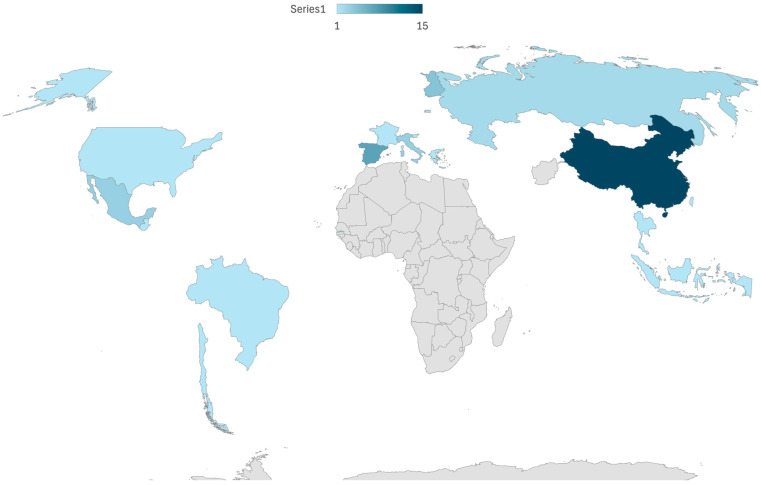
Map of the represented countries.

The 46 studies investigated colostrum samples from 2352 mothers (Table 1) [17,28,29,30,31,32,33,34,35,36,37,38,39,40,41,42,43,44,45,46,47,48,49,50,51,52,53,54,55,56,57,58,59,60,61,62,63,64,65,66,67,68,69,70,71]. The studies were conducted across 17 countries, with the majority (18) taking place in a single country, and one study conducted in two countries. The countries represented include China (15 studies), Spain (7 studies), Finland (4 studies), Mexico (3 studies), Russia (2 studies), Italy (3 studies), and one study each in Guatemala, Brazil, Burundi, Chile, France, Gambia, Greece, Indonesia, Slovenia, Taiwan, Thailand, Switzerland, and the USA. A comprehensive map of the represented countries is presented below (Figure 2).

Among the 46 studies, 5 studies specifically targeted lactic acid bacteria [36,39,51,53,72], one study examined bacteria typically found in the mother’s gut [55], one study analyzed the presence of fungi [29], and one study investigated methanogenic archaea [17]. A variety of methodologies were employed to analyze the microbiota, including bacterial culture (1 study), polymerase chain reaction (PCR) (5 studies), matrix-assisted laser desorption ionization-time of flight mass spectrometry (MALDI-TOF-MS) (3 studies), 16S rRNA gene sequencing (15 studies), and metagenomic shotgun sequencing (1 study). Eleven (11) studies used two methods, and four studies employed three different methods for microbiota analysis.

Regarding the DNA extraction methods, 15 different DNA extraction kits were utilized. The sequencing was performed using seven different sequencing machines, including Genome Sequencer FLX, Ion Torrent PGM, Illumina MiSeq, Illumina HiSeq 2500, Illumina NovaSeq 6000, Illumina HiSeq 3000, and the PacBio Sequel sequencer. These studies targeted five different hypervariable regions (V1–V3, V3, V3–V4, V4, V3–V5) and used nine different databases for taxonomic classification, including Ribosomal Database Project (RDP), Greengenes, Silva, NCBI BLAST, Kraken2, RefSeq, Myla Database (BioMérieux), GenBank, and MicroSEQ ID.

Information on study design and results regarding each of the included studies can be found in Table 2.

### 3.1. General Composition

#### 3.1.1. Bacteria

The bacterial diversity in colostrum varied across studies, with up to 465 genera, 645 species, and 7900 operational taxonomic units (OTUs) detected [57,71]. The number of OTUs identified per colostrum sample ranged from 122 to 341 [55,57]. Bacterial loads reported by culture-based methods ranged from 3.7 × 10^2^ to 1.1 × 10^9^ genome equivalents (GE) per mL [54,70], while qPCR methods detected bacterial loads between 10^4^ and 10^6^ CFU/mL [28,29].

Regarding bacterial abundance and diversity, Khodayar-Pardo et al. reported that colostrum has the lowest total bacterial count, with a progressive increase in total bacteria and specific groups like *Bifidobacterium* and *Enterococcus* spp. across lactation, leading to a more enriched microbial profile in mature milk [48]. Similarly, Boix-Amorós et al. observed lower diversity in colostrum and a peak in bacterial diversity during transition milk, though they noted a greater abundance of *Acinetobacter* in colostrum, while *Staphylococcus* remained dominant across all lactation stages [29]. Conversely, other studies highlighted higher bacterial abundance in colostrum. Cabrera-Rubio et al. reported that colostrum exhibits higher diversity compared to 1-month and 6-month milk samples, suggesting a decline in bacterial diversity as milk matures [32]. Collado et al. reported higher counts of *Enterococcus* in colostrum, whereas mature milk showed increased abundance of *Staphylococcus aureus* and *Clostridium coccoides*, indicating a shift in microbial composition over time [34]. Similarly, Xie et al. emphasized the greater heterogeneity and a notable presence of environmental bacteria in colostrum, suggesting elevated microbial abundance during this early stage of lactation [71]. In line with this, Liu et al. reported that microbial diversity increases as milk progresses from colostrum to mature milk, with *Firmicutes* and *Proteobacteria* as the dominant phyla and core genera such as *Streptococcus*, *Staphylococcus*, *Serratia*, and *Corynebacterium*, highlighting the dynamic nature of microbial composition across lactation [50]. Additionally, Qi et al. observed that colostrum exhibits a higher abundance of specific genera such as *Bifidobacterium*, *Fusicatenibacter*, and *Blautia*, which decrease as lactation progresses, while genera like *Staphylococcus* remain prominent throughout all stages [55].

The predominant phyla in colostrum were *Firmicutes* and *Proteobacteria*, while *Actinobacteria* and *Bacteroidetes* were less abundant [49,57,61,66,67]. Additional phyla identified included *Acidobacteria*, *Armatimonadetes*, *Chlamydiae*, and *Verrucomicrobia* [28,29,31,57]. Core genera consistently identified across studies included *Bifidobacterium* spp., *Staphylococcus*, and *Streptococcus*, with *Bifidobacterium longum* and *Bifidobacterium breve* detected in nearly all samples [28]. Other genera commonly found in colostrum included *Staphylococcus*, *Acinetobacter*, *Pseudomonas*, *Lactobacillus*, and *Clostridium* clusters XIVa-XIVb [29,31]. Wyatt et al. reported high prevalence of *Streptococcus* and *Micrococci*, while Tang et al. and Obermajer et al. observed the presence of *Enterobacteriaceae*, including *Acinetobacter* and *Stenotrophomonas* [54,60,70].

Several studies showed that colostrum contains higher bacterial diversity compared to both maternal and infant feces. Cabrera-Rubio et al. reported that colostrum harbored greater diversity than both infant and maternal feces [31]. Singh et al. noted that colostrum contained a higher number of amplicon sequence variants (ASVs) than infant feces, while Qi et al. highlighted significant bacterial transfer from colostrum to infant feces [55,57]. Xie et al. also confirmed differences in microbiota composition between colostrum and infant feces [71].

#### 3.1.2. Probiotic Bacteria

Damaceno et al. reported the presence of *Lactobacillus gasseri* and *Bifidobacterium breve* in colostrum samples, with higher prevalence in vaginal deliveries and mothers with normal BMI [36]. Dubos et al. reported that 55.3% of colostrum samples were positive for *Lactobacillus*, with *Lactobacillus plantarum* being the most abundant species (64% of isolates), followed by *Lactobacillus fermentum* (16%) [42]. Martin et al. identified *Lactobacillus gasseri* and *Lactobacillus fermentum* in breast milk isolates from mother–infant pairs, as well as *Enterococcus faecium* in both breast milk and infant feces [19]. Nikolopoulou et al. detected *Bifidobacterium* in 61.5% of colostrum samples, with *Bifidobacterium longum* and *Bifidobacterium bifidum* as the most prevalent species [53]. *Lactobacillus* was found in 46.2% of samples, with both *Bifidobacterium* and *Lactobacillus* detected in 76.9% of colostrum samples.

#### 3.1.3. Microbes Other than Bacteria

Boix-Amorós et al. reported a median fungal load in colostrum of 4.1 × 10^5^ cells/mL, with similar levels observed in transitional milk (4.5 × 10^5^ cells/mL) and lower levels in mature milk (2.8 × 10^5^ cells/mL). Fungal DNA was detected in 16 of the 18 colostrum samples, with dominant fungal genera including *Malassezia*, *Candida*, and *Saccharomyces*. *Malassezia* was found in all colostrum samples where fungi were detected but was not isolated in culture [30].

Togo et al. analyzed 20 samples from 13 mothers for methanogenic archaea, with positive cultures in 9 out of 20 samples (3 colostrum and 6 milk samples). *M. smithii* was isolated in eight samples from five mothers, while *M. oralis* was isolated in one milk sample. Real-time PCR detection in 136 samples revealed M. smithii in 27.3% of colostrum samples (32/117) and 26.3% of milk samples (5/19), with a median DNA concentration of 463 copies/mL in colostrum [17].

### 3.2. Factors That Influence Breast Milk Microbiota

#### 3.2.1. Diet

Dietary factors can influence the composition of colostrum microbiota, and studies focus on the effects of macronutrients, micronutrients, and specific dietary patterns. Drago et al. found differences in the microbial hubs of colostrum between Italian and Burundian mothers, likely due to dietary differences between them. The Italian diet, rich in animal proteins, fats, and sugars, was associated with bacterial hubs like *Abiotrophia* spp. and *Parabacteroides* spp., while the Burundian plant-based diet resulted in bacterial hubs such as *Aquabacterium* spp. and *Rhizobium* spp., reflecting the influence of fiber-rich foods on the microbiome [40].

Nikolopoulou et al. reported that women who consumed yogurt regularly had a higher prevalence of *Lactobacillus* and *Bifidobacterium* in their colostrum, suggesting that probiotic intake through diet could contribute to these bacterial populations [53]. Williams et al. observed that maternal intake of saturated and monounsaturated fatty acids was negatively associated with *Corynebacterium*, while carbohydrate intake (lactose and disaccharides) was negatively associated with *Firmicutes* in colostrum [68]. Additionally, micronutrients such as pantothenic acid, riboflavin, and calcium were associated with *Veillonella* and *Streptococcus*, highlighting the impact of micronutrient intake on microbiota composition.

Tapia Gonzalez et al. [62] reported that non-nutritive sweeteners (NNS) did not significantly affect the diversity of colostrum microbiota, although some shifts were observed in specific genera, such as an increase in *Bifidobacterium* and a decrease in *Staphylococcus* and *Blautia* with higher NNS consumption. Tang et al. [60] noted that Hexachlorocyclohexane (HCH), a persistent organic pollutant commonly found in fish, was associated with changes in microbial diversity, with *Pseudomonas* and *Enterococcus* showing notable alterations in colostrum from high HCH exposure groups.

#### 3.2.2. Maternal Body Mass Index

Maternal BMI influences the microbiota composition in colostrum. Cabrera-Rubio et al. and Collado et al. observed higher bacterial counts of *Lactobacillus* and *Staphylococcus* in colostrum from overweight or obese mothers compared to that from mothers with normal BMI [31,34]. Cabrera-Rubio et al. further reported a reduction in bacterial diversity in colostrum from obese mothers, which diminished in later lactation stages [31]. Similarly, Dave et al. found that a higher maternal pre-pregnancy BMI correlated with a lower relative abundance of *Streptococcus* but higher overall microbial diversity in breast milk [37].

Studies showed that *Bifidobacterium* and *Lactobacillus* detection in colostrum decreases with increasing maternal BMI. Nikolopoulou et al. reported that *Lactobacillus* positivity dropped significantly in mothers with higher BMI [53]. Additionally, Togo et al. reported that the prevalence of *M. smithii* was lower in obese mothers compared to lean or overweight mothers [17].

#### 3.2.3. Delivery Mode

Delivery mode influences the microbial composition of colostrum, and several studies highlight differences between vaginal and cesarean delivery in terms of bacterial diversity and composition. Cabrera-Rubio et al. and Khodayar-Pardo et al. reported that colostrum from cesarean deliveries had higher total bacterial concentrations and altered bacterial composition compared to vaginal deliveries, with an increased abundance of certain families such as *Carnobacteriaceae* and *Streptococcus* spp., while *Bifidobacterium* spp. was more frequently detected in vaginal deliveries [31,48].

Toscano et al. noted that colostrum from vaginal delivery had slightly higher biodiversity, with significantly higher levels of *Streptococcus* and *Haemophilus*, whereas cesarean delivery colostrum showed increased levels of *Finegoldia*, *Halomonas*, *Prevotella*, *Pseudomonas*, and *Staphylococcus* [63]. Additionally, Mastromarino et al. found that vaginal delivery was associated with significantly higher levels of *Lactobacilli* and *Bifidobacteria* in colostrum, whereas cesarean delivery showed no such increase [51].

In terms of microbial networks, Toscano et al. identified distinct bacterial hubs for both delivery modes, with *Ruminococcus* and *Peptostreptococcus* being key nodes in cesarean colostrum, while *Rhodanobacter* and *Achromobacter* were central in vaginal delivery colostrum [63]. Similarly, Xie et al. reported that cesarean delivery colostrum exhibited higher diversity compared to vaginal delivery, with distinct microbial profiles identified through principal component analysis (PCA) [71].

#### 3.2.4. Gestational Age

Gestational age influences the microbiota composition of breast milk, with notable differences between preterm and term samples. Du et al. reported that gestational age did not significantly affect alpha or beta diversity in breast milk [41]. However, Khodayar-Pardo et al. observed that *Bifidobacterium* spp. levels were higher in milk from full-term infants across all stages of lactation, with significant differences noted in colostrum, transitional milk, and mature milk (*p* = 0.003, *p* = 0.005, *p* = 0.014), while *Enterococcus* spp. levels were lower in term milk, particularly colostrum (*p* = 0.045) [48].

Moles et al. reported higher bacterial counts in mature milk compared to colostrum for extremely preterm infants, with significantly higher frequencies of *Enterococci* (*p* = 0.000), Lactobacilli (*p* = 0.041), and *Enterobacteria* (*p* = 0.038) in mature milk [52]. Additionally, Singh et al. found that preterm breast milk had higher microbial richness and diversity compared to term milk, with significant differences in the *Actinobacteria* and *Bacteroidetes* phyla. Genera such as *Faecalibacterium*, *Prevotella*, *Clostridium*, *Bacteroides*, and *Enterobacter* were more abundant in preterm samples, whereas term milk showed higher levels of OD1 phyla and enrichment in *Staphylococcus epidermidis*, unclassified *Veillonella*, and unclassified OD1 species [57].

#### 3.2.5. Human Milk Oligosaccharides (HMOs)

HMOs in colostrum influence microbial composition. Aakko et al. reported a positive correlation between total HMO concentration and the presence of *Bifidobacterium* spp. (ρ = 0.63, *p* = 0.036), and sialylated HMOs strongly correlated with *Bifidobacterium breve* (ρ = 0.84, *p* = 0.001) while fucosylated HMOs correlated with *Akkermansiamuciniphila* (ρ = 0.70, *p* = 0.017). Sun et al. observed significant correlations between specific HMOs and bacterial taxa: *Lactobacillus* was positively correlated with LNT (r = 0.250, *p* = 0.037), while *Staphylococcus* was negatively correlated with DS-LNT (r = −0.240, *p* = 0.045). *Streptococcus* in colostrum showed positive correlations with LNFP II, LNFP III, and 3-SL (*p* < 0.05), suggesting that HMOs modulate the presence of specific bacterial taxa [59,73]. Additionally, differences in HMO profiles were observed based on maternal characteristics: non-fucosylated HMOs were lower in milk from mothers who delivered vaginally compared to cesarean deliveries (*p* = 0.038), while fucosylated HMOs were higher in primipara compared to multipara mothers (*p* = 0.030). Cabrera-Rubio et al. further highlighted that the presence of certain HMOs, such as 2′FL, was associated with higher levels of Lactobacillus in secretor colostrum, while Ge et al. reported that *Staphylococcus* and *Gemella* were negatively correlated with several HMOs in colostrum, including 3′-SL, LNT2, LSTc, and total HMOs. In contrast, *Serratia*, *Pseudomonas*, and *Stenotrophomonas* showed significant positive correlations with multiple HMOs, especially LNT2 and total HMOs [32,45].

#### 3.2.6. Maternal Secretor Status

Maternal secretor status influences the microbiota composition in colostrum. Cabrera-Rubio et al. found that colostrum from secretor mothers had significantly higher levels of *Lactobacillus* spp. (*p* = 0.0004), *Streptococcus* spp. (*p* = 0.0030), and *Enterococcus* spp. (*p* = 0.014) compared to non-secretors. However, *Bifidobacterium* spp. levels were lower in secretor colostrum (*p* = 0.040). Secretor colostrum also had higher concentrations of 2′-fucosyllactose (2′FL) and lacto-N-fucopentaose I (LNFP I), while non-secretor colostrum contained higher levels of LNFP II and lacto-N-difucohexaose II (LNDFH II). Additionally, a positive association between higher *Lactobacillus* levels and 2′FL was observed (Spearman’s rho = 0.542, *p* = 0.038) [32].

#### 3.2.7. Maternal Age

Maternal age influences the microbiota composition in human milk, though the effects are not always consistent across studies. Corona-Cervantes et al. reported a slight tendency for a decrease in milk richness (Chao1) with increasing maternal age, while diversity (Shannon) tended to increase with the number of days postpartum [35]. Nikolopoulou et al. observed a decline in the presence of *Lactobacillus* and *Bifidobacterium* in milk samples as maternal age increased, with 100% of women aged 18–29 having positive samples, compared to only 26.1% in women aged 35–39 and 0% in women aged 45 and older [53]. However, Wan et al. found no statistically significant differences in microbial diversity or richness between mothers aged ≤ 30 and >30 (*p* = 0.35 for diversity and *p* = 0.79 for richness) [65].

#### 3.2.8. Macronutrients of Colostrum and Fungi

Boix-Amorós et al. found significant correlations between fungal load and milk components, suggesting that certain macronutrients might influence fungal populations in breast milk. Fungal load was positively correlated with fat content and non-fatty solids (NFS), indicating that both fat and specific non-fatty nutrients may support fungal presence. Specific fungal genera also showed associations with milk components: *Malassezia* displayed a strong positive correlation with bacterial load (Spearman’s ρ = 0.93, *p* = 0.007) and lactose content (ρ = 0.78, *p* = 0.048), while Candida was positively correlated with protein content (ρ = 0.77, *p* = 0.044), suggesting that protein may specifically support this genus. In contrast, *Lodderomyces* exhibited a negative correlation with human somatic cells (ρ = −0.79, *p* = 0.035), indicating an inverse relationship between immune cell concentrations and fungal presence [30].

##### IL-6

Collado et al. reported that higher IL-6 levels correlated with higher *Staphylococcus* counts in normal-weight mothers (r = 0.628, *p* = 0.039). However, in overweight mothers, higher IL-6 levels were associated with lower *Akkermansiamuciniphila* counts (r = −0.738, *p* = 0.015), suggesting that IL-6 may play a role in modulating the microbiota composition of colostrum, with distinct effects depending on maternal weight status [34].

#### 3.2.9. Probiotics During Pregnancy

Two studies (70 and 66 participants) investigated the effects of probiotic supplementation during pregnancy on the microbiota in colostrum. Dewanto et al. reported that *Bifidobacterium animalis* lactis HNO19 (DR10) was detected in 14% of mothers in the probiotic group at birth, increasing to 20% at 3 months postpartum, while the placebo group showed no detectable DR10. However, no significant differences were found in total microbiota or in *Lactobacillus* and *Bifidobacterium* levels between the groups at either time point [39]. In contrast, Mastromarino et al. observed that the probiotic-supplemented group had significantly higher counts of *Lactobacilli* (4.5 × 10^3^ cells/mL) and *Bifidobacteria* (1.7 × 10^4^ cells/mL) in colostrum compared to the placebo group [51].

#### 3.2.10. Geographic Location

Geographic location also influences the composition of colostrum microbiota, with differences noted in bacterial diversity and specific bacterial hubs between regions. Nikolopoulou et al. found a higher prevalence of *Lactobacillus* and *Bifidobacterium* in colostrum from rural areas compared to urban areas, with 97.1% of rural samples testing positive vs. 33.8% in urban samples [53]. Similarly, Wan et al. reported significant differences in microbial diversity and richness across cities, with genus-level variations in colostrum microbial composition [65]. In terms of bacterial hubs, Drago et al. identified *Aciditerrimonas* spp. as the central node in Italian colostrum, while *Aquabacterium* spp. was the central node in Burundian colostrum, reflecting differences driven by environmental exposure and dietary influences [40].

#### 3.2.11. Perinatal Antibiotic Exposure

Perinatal antibiotic exposure has varying effects on breast milk microbiota composition. Du et al. and Ji et al. reported no significant impact on microbial diversity or composition in breast milk following intrapartum or post-delivery antibiotic treatment (Cefuroxime (CXM), Ceftriaxone (CFX)) [41,46]. However, specific shifts were observed; for instance, Du et al. noted enrichment of *Lachnospiraceae* in the milk of mothers who received Intrapartum antibiotic prophylaxis (IAP), while Ji et al. found *Pseudomonas* and *Clostridiaceae* to be more abundant in certain antibiotic-treated groups. In contrast, Wang et al. observed significant changes in colostrum microbiota after post-delivery antibiotic treatment, with decreased alpha diversity and reductions in genera like *Actinomyces* and *Clostridium* [66]. Singh et al. reported no significant changes in colostrum microbiota associated with antibiotic use during delivery [57].

#### 3.2.12. Gender of the Neonate

The gender of the neonate influences the composition of breast milk microbiota in some studies. Du et al. reported no significant differences in alpha and beta diversity based on infant gender; however, specific genera varied, with *Streptococcaceae* enriched in the milk of mothers with female infants, while *Roseburia* and *Alcaligenaceae* were more abundant in the milk of mothers with male infants [41]. Similarly, Gamez-Valdez et al. observed gender-related differences in microbial composition in the context of obesity and gestational diabetes. Higher levels of *Gemella* and *Staphylococcus* were detected in the obesity-female group, while *Prevotella* and *Rhodobacteraceae* were enriched in the GDM-female subgroup [44]. Additionally, Williams et al. found that milk from mothers of male infants had higher levels of *Streptococcus* and lower levels of *Staphylococcus* compared to milk from mothers of female infants [68].

#### 3.2.13. Maternal Stress

In two studies, maternal stress was found to affect the composition of human milk microbiota [38,43]. Fernández-Tuñas et al. reported that milk from low-stress mothers had higher microbial diversity (Shannon index of 1.19) compared to milk from high-stress mothers (0.99). In high-stress mothers, *Proteobacteria* increased from 12.5% on day 3 to 44.4% on day 15, while *Firmicutes* decreased from 87.5% to 55.6%. In contrast, for low-stress mothers, *Firmicutes* remained dominant with a slight increase in *Proteobacteria*. Other phyla, such as *Actinobacteria* and *Bacteroidetes*, were rarely detected [43]. Respectively, Deflorin et al. reported that prenatal generalized anxiety and postpartum anxiety both negatively correlated with the colostrum microbiome, although there was no association between maternal mental health scores and alpha or beta diversity (false discovery rate (FDR) > 0.05) [38].

#### 3.2.14. Gestational Diabetes Mellitus (GDM)

Gámez-Valdez et al. reported that *Staphylococcus* was more abundant in GDM mothers, with levels higher in both the GDM-female group (15.6%) and the GDM-male group (21.9%) compared to controls. *Prevotella* was also more abundant in GDM mothers (6.1% in GD-F and 6.8% in GD-M) than in healthy controls (4.8%), with higher levels in females. Both *Corynebacterium* and *Anaerococcus* were significantly enriched in GDM colostrum compared to controls. GDM colostrum exhibited higher alpha diversity than controls, with GDM-females showing greater diversity than GDM-males. Distinct taxonomic differences were also noted, including increased *Burkholderia* in the obesity-female group and *Rhodobacteraceae* and *Xanthobacteraceae* in the GDM-female group [44].

#### 3.2.15. Feeding Type

Feeding type influences the relationship between maternal milk and infant gut microbiota, based on Li et al. who found that in the breastfeeding (BF) group, there were more consistent and specific correlations between maternal milk and infant gut microbiota. In contrast, the mixed feeding (MF) group displayed a greater variety of species correlated between breast milk and the infant’s gut, indicating that mixed feeding introduces additional variability to microbiota. This suggests that breastfeeding may support a more stable microbial environment, while mixed feeding introduces greater diversity [49].

#### 3.2.16. Milk Collection Methods

The way of milk collection influences the bacterial load and composition of breast milk microbiota. Sakwinska et al. compared two collection protocols: a standard protocol and an aseptic protocol. The standard protocol showed a significantly higher total bacterial load compared to the aseptic protocol, with a median of 7.5 × 10^4^ counts/mL vs. 7.8 × 10^3^ counts/mL at 5–11 days postpartum (*p* < 0.0001). However, this difference was not significant at earlier or later time points. *Bifidobacteria* and *Lactobacilli* were detected in very few samples, with slightly higher proportions in the aseptic protocol. Performing 16S rRNA gene sequencing revealed more bacterial DNA in standard protocol samples, with *Staphylococcus* and *Streptococcus* making up 42% and 40% of the microbiota, respectively, in both protocols. In contrast, aseptic samples showed a significantly lower abundance of *Acinetobacter* (1.8%) compared to standard protocol samples (32%). Multivariate analysis confirmed significant differences in microbiota composition between the two collection methods, underscoring the impact of collection protocol on the milk microbiota profile [56].

#### 3.2.17. Mastitis

Tao et al. compared colostrum from healthy women and women with mastitis, highlighting key differences. In healthy women, colostrum had significantly higher levels of *Lactobacillus* and *Bifidobacterium* compared to *Staphylococcus* and *Streptococcus* (*p* < 0.0001). In mastitis milk (from both affected and unaffected breasts), these beneficial bacteria were significantly lower. In mastitis cases caused by *Staphylococcus aureus* (S.A.) or Methicillin-resistant *Staphylococcus aureus* (MRSA), Staphylococcus levels in colostrum were lower than in the affected breast but similar to the unaffected breast. However, in *Staphylococcus epidermidis* (S.E.) or *Staphylococcus lentus* (S.L.)-mediated mastitis, *Staphylococcus* levels in colostrum were similar to or higher than in mastitis milk. *Bifidobacterium* levels remained higher in colostrum than in both affected and unaffected breasts of Staphylococcus-related mastitis patients (*p* < 0.01). In non-bacterial mastitis cases, colostrum showed significantly higher bacterial levels compared to mastitis milk (*p* < 0.01). Cytokine analysis revealed significantly higher levels of IL-6 and IL-8 in mastitis milk compared to colostrum, while CRP, TNF-α, and IL-1 levels showed no significant differences between the two groups [61].

#### 3.2.18. HPV Infection

HPV infection was detected in both breast milk and infant oral samples, and Tuominen et al. reported that 8.6% of mothers’ breast milk samples tested positive for HPV DNA, compared to 40% of infant oral samples. The bacterial composition in colostrum revealed that *Firmicutes* (79.7%), *Proteobacteria* (14.1%), *Actinobacteria* (5.2%), and *Bacteroides* (0.5%) were the predominant phyla. Notably, *Proteobacteria* was significantly more abundant in breast milk compared to the infant oral cavity (4.5%, *p* = 0.0082), while *Bacteroides* was more abundant in the infant oral cavity (6.0%, *p* = 0.009). The shared core microbiota between breast milk and the infant oral cavity included genera such as *Streptococcus*, *Staphylococcus*, *Rothia*, and *Veillonella* [64].

Although no significant differences in species richness, diversity, or microbial composition were found between HPV-positive and HPV-negative breast milk samples, breast milk showed higher microbial diversity, compared to the infant oral cavity (*p* = 0.0043, Shannon index), and distinct clustering patterns (*p* = 0.0001, RDA). Additionally, HPV-positive infant oral samples exhibited a distinct clustering pattern compared to HPV-negative samples (*p* = 0.036, RDA). Levels of *Veillonella* dispar were significantly higher in HPV-negative infant oral samples at both the genus (*p* = 0.025) and species (*p* = 0.048) levels [64].

#### 3.2.19. Gestational Hypertension

Gestational affects the microbial composition in breast milk, particularly in mothers with gestational prehypertension. Wan et al. observed that mothers with gestational hypertension had lower microbial diversity and richness in their colostrum compared to normotensive mothers, with significant differences (*p* < 0.05). Additionally, *Lactobacillus* abundance in colostrum was lower in prehypertensive mothers, with a trend towards significance in colostrum (*p* = 0.09) and a significant decrease in transitional milk (*p* = 0.004) [65].

#### 3.2.20. Ethnicity

Ethnicity influences the microbial composition of breast milk. Xie et al. compared colostrum microbiota between Li and Han ethnic groups, noting significant differences at both phylum and genus levels. In the Li ethnic group, *Proteobacteria* (66.5%) was the dominant phylum, followed by *Firmicutes* (29.5%), while in the Han ethnic group, *Firmicutes* (46.5%) was most dominant, followed by *Proteobacteria* (43.7%). At the genus level, *Cupriavidus* (26.28%), *Staphylococcus* (17.36%), and *Streptococcus* (13.11%) were the most abundant in the Li group, while *Acinetobacter* (28.72%), *Staphylococcus* (28.38%), and *Streptococcus* (9.45%) dominated in the Han group [71].

Species-level analysis revealed differences in abundance, with *Cupriavidus lacunae* and *Streptococcus himalayensis* being more abundant in the Li group, and *Staphylococcus petrasii* and *Acinetobacter proteolyticus* being more prominent in the Han group. Additionally, Han mothers exhibited higher alpha diversity (Chao1 and Ace indices) compared to Li mothers. Beta-diversity analysis showed a distinct separation between the two ethnic groups. Differences were also noted in the core microbiota, with *Cupriavidus lacunae* and *Streptococcus himalayensis* significantly abundant in the Li group, and *Staphylococcus petrasii* and *Acinetobacter proteolyticus* more prevalent in the Han group. Delivery mode further influenced the microbiota, with Han mothers sharing a core microbiota of *Acinetobacter*, *Staphylococcus*, and *Streptococcus* between cesarean and vaginal deliveries, while Li mothers shared genera such as *Cupriavidus*, *Enterococcus*, and *Streptococcus* [71].

#### 3.2.21. Parity

Parity was reported to have no significant effect on the diversity of breast milk microbiota. Du et al. found that both alpha and beta diversity measures showed no significant differences between milk samples from mothers with varying numbers of pregnancies [41].

#### 3.2.22. Entero-Mammary Pathway

The concept of the entero-mammary pathway proposes that maternal gut bacteria can translocate to the mammary glands and colonize breast milk, including colostrum [41]. In the study by Du et al., colostrum exhibited greater microbial abundance and a distinct composition compared to nipple skin, with 111 unique OTUs, supporting the hypothesis of an entero-mammary pathway. Anaerobes such as *Bifidobacterium*, *Pantoea*, and *Enterobacteriaceae* were mainly identified in colostrum, while *Staphylococcus*, *Bacteroides*, and *Parabacteroides* dominated the nipple skin. Infant sex, gestational age, and maternal factors (e.g., delivery mode, IAP) showed limited influence on diversity, though cesarean delivery and full-term birth were associated with increased *Bifidobacteria* [41].

## 4. Discussion

The results of our study indicate that colostrum microbiota is diverse and predominantly consists of genera such as *Staphylococcus*, *Streptococcus*, *Lactobacillus*, *Bifidobacterium*, and *Enterococcus*, with significant presence of *Acinetobacter*, *Prevotella*, *Corynebacterium*, and *Rhodobacteraceae*. Additionally, the presence of other microbial groups, including fungi and archaea, was observed.

Based on the studies included in this review, there is evidence suggesting that factors such as maternal body mass index (BMI), delivery mode, gestational diabetes mellitus (GDM), feeding type, and geographic location influence the composition of colostrum microbiota. Additionally, maternal stress, perinatal antibiotic exposure, maternal age, and maternal secretor status were also identified as potential factors affecting the microbiota. However, many of the studies were small and the results varied across different populations and conditions. Therefore, caution should be exercised in drawing definitive conclusions about the relative impact of these factors on the composition of colostrum microbiota.

Various mechanisms were described regarding the mechanism by which microbes reach and colonize colostrum. One such mechanism involves retrograde flow, where breast milk (BM) flows back into the mammary ducts, allowing bacteria to enter the mammary glands [74]. Studies, such as Cortez et al., suggest that BM may contribute to the colonization of the infant’s oral cavity, as buccal administration of colostrum altered the oral microbiota in low-birth-weight infants [75]. Additionally, bacteria were detected in colostrum before suckling, indicating that microbes are present in the milk prior to the infant’s first feed [36]. Maternal skin microbiota is also a possible source of colostrum microbes, with skin-associated bacteria like *Staphylococcus*, *Cutibacterium*, and *Corynebacterium* found in BM [76]. Moreover, the entero-mammary pathway, where gut bacteria migrate to the mammary glands, is thought to play a part, particularly for anaerobic bacteria such as *Bifidobacterium* [76]. Finally, bacterial translocation, a physiological event heightened during pregnancy and lactation as described in rodents, may also contribute to the influx of bacteria into colostrum [77]. These mechanisms highlight the multifaceted nature of microbial colonization in colostrum.

Several studies reported higher diversity in colostrum microbiota compared to mature breast milk. Khodayar-Pardo et al. found greater microbial diversity in colostrum, while Xie et al. noted higher microbial richness in colostrum, although no significant differences in diversity indices were observed [48,71]. Boix-Amoros et al. found no significant differences across colostrum, transitional milk, and mature milk, but highlighted that non-viable bacteria and extracellular DNA could affect qPCR results, suggesting that viable bacterial numbers may be lower than reported [29]. These findings suggest that colostrum is a more diverse source of microorganisms. The increased microbial diversity in colostrum is likely due to the open tight junctions of the mammary gland epithelium in the early days post-birth, allowing the transport of immune cells into the mammary gland [76]. High leucocyte levels in colostrum reflect the need for immunological protection in the newborn. These immune cells are closely associated with a significant proportion of milk bacteria, influencing the milk microbiota composition. As lactation progresses, immune cell populations decrease, causing changes in milk microbiota composition [4].

Some studies reported that colostrum contains a higher bacterial diversity compared to both maternal and infant feces [31,57]. Xie et al. highlighted the significant transfer of bacteria from colostrum to infant feces, further confirming differences in microbiota composition between the two [71]. This raises the following question: why is it important to present such a diverse colostrum microbiota? Boix Amoros et al. suggests that no correlation between human and bacterial cells was found in milk, indicating that the milk microbiota is not perceived as an infection by the mother’s immune system [29]. Instead, the immune response is directed toward specific microorganisms like *Staphylococcus*, without triggering an inflammatory response in the mammary ducts. The presence of lactic acid bacteria (LAB) in colostrum is of particular interest due to their antimicrobial properties. LAB produce antimicrobial peptides, such as bacteriocins, which are considered promising natural preservatives and therapeutic agents [78,79]. LAB inhibit the growth of harmful pathogens such as *Bacillus cereus*, *E. coli*, *E. faecalis*, *Listeria monocytogenes*, *P. aeruginosa*, *S. aureus*, and *Salmonella* serotype *Enteritidis* [80]. Moreover, *Weissella*, a genus of LAB, inhibits biofilm formation and exhibits anti-inflammatory effects, further highlighting the protective role of colostrum microbiota in infant health [81].

In addition to antimicrobial properties, the colostrum microbiota provides several health benefits for both the infant and the mother. Research by Suarez Martinez et al. indicates that colostrum contains diverse bacteria, such as *Lactobacillus*, *Bifidobacterium*, and *Streptococcus*, which are essential for the early colonization of the infant gut [82]. These bacteria aid in digestion and contribute to the maturation of the immune system. Furthermore, studies showed that the microbiota in colostrum is more diverse than that in mature milk, with genera like *Weissella* and *Leuconostoc* being more prevalent in colostrum. As a result, breastfed infants have a gut microbiota that is less diverse but richer in beneficial bacteria like *Bifidobacterium* and *Lactobacillus*, which are associated with a lower risk of allergies and improved immune function [83,84]. Bifidobacterium, for example, releases short-chain fatty acids (SCFAs) such as butyrate and propionate, which lower stool pH and create an unfavorable environment for pathogenic bacteria, reducing the risk of food allergies [85].

The composition of the breast milk microbiota also plays a role in maternal health. Heikkla et al. found that species such as *S. epidermidis*, *S. salivarius*, *E. faecalis*, *Lactobacillus*, *Lactococcus*, and *Leuconostoc* suppress the growth of *S. aureus*, a major cause of mastitis [86]. Differences in the milk microbiota could explain why some women experience recurrent mastitis while others do not, suggesting that a healthy and diverse milk microbiota may help protect mothers from infections like mastitis [82].

Several maternal and environmental factors appear to influence the composition of breast milk microbiota. The stage of lactation plays a central role, with colostrum generally showing higher bacterial diversity compared to transitional and mature milk [29,48]. Mode of delivery has also been consistently associated with microbial differences, with vaginal birth favoring the transfer of maternal vaginal and gut microbes, whereas cesarean section is linked to altered microbial communities in milk [31,49]. Maternal characteristics, including BMI and GDM, further shape microbial profiles, with overweight mothers and those with GDM showing reduced bacterial diversity and altered abundance of genera such as *Staphylococcus* and *Lactobacillus* [31]. Geographic location and maternal diet also appear to contribute, as differences appear in milk microbiota composition between women in Europe, Africa, and Asia, suggesting the influence of environmental exposures and nutritional patterns [40,65].Additional influences include maternal age, parity, perinatal antibiotic use, infant sex, and even milk collection method, which may introduce external bacteria [41,56].

Our study has limitations. Firstly, most studies included in this review were designed as observational cohorts, which are prone to residual confounding. Secondly, the heterogeneity among included studies regarding study design, sample size, data collection methods, and analysis limited the ability to perform a meta-analysis and made direct comparisons challenging. Also, some studies had small sample sizes, which may affect the reliability of the findings. Lastly, language restrictions may have led to the exclusion of relevant studies.

## 5. Conclusions

In summary, colostrum contains a highly diverse microbiota, predominantly comprising genera such as *Staphylococcus*, *Streptococcus*, *Lactobacillus*, *Bifidobacterium*, and *Enterococcus*, along with other microbial groups including fungi and archaea. The composition and diversity of this microbiota are shaped by a range of intrinsic factors, such as maternal body mass index (BMI), age, secretor status, stress levels, and health conditions like gestational diabetes, as well as extrinsic factors, including delivery mode, antibiotic exposure, feeding practices, and geographic location.

Colostrum microbiota plays a pivotal role in both infant and maternal health. In infants, it promotes early gut colonization, aids digestion, supports immune system maturation, and provides protection against pathogens through mechanisms such as the production of antimicrobial peptides by lactic acid bacteria. In mothers, a diverse milk microbiota may help prevent infections such as mastitis and contribute to overall mammary gland health.

This microbiota plays a crucial role in promoting maternal and infant health, particularly in the early stages of life. Interventions aimed at enhancing the beneficial properties of colostrum or improving its microbial composition could offer significant opportunities to positively impact infant health, especially in promoting gut health and immune development. Further research with larger, well-designed studies is needed, focusing on the identification of the full range of microbes in colostrum, including fungi, archaea, and viruses, and exploring the interactions between these microbes and their potential effects on infant health.

## Figures and Tables

**Table 1:** Study Characteristics.

First Author	Year	Country	Study Design	Study Population; N	Milk Samples; N	Aim
Deflorin [39]	2025	Switzerland	Observational study	100	91 pp	To investigate how maternal mental health relates to the human milk microbiome
Ge [45]	2024	China	Observational Study	40	40 pp	To compare the human milk microbiota and oligosaccharide profiles between colostrum and mature milk
Sun [59]	2024	China	Pilot study	70	70 in 5 d pp	To investigate the associations between human milk oligosaccharide concentrations and clinical factors as well as correlations with specific microbial taxa in colostrum
Fernández-Tuñas [43]	2023	Spain	Prospective observational study	45	45 in 3 d pp, 45 in 7 d pp, 45 in 15 d pp	To investigate the impact of maternal perinatal stress on mothers’ own milk production and microbiota, as well as the intestinal microbiota of very preterm newborns
Singh [57]	2023	Thailand	Comparative observational study	48	96 in 0–3 d pp, 96 in 7–15 d pp, 96 in 2 mo pp	To investigate the impact of preterm birth on the composition and diversity of breast milk microbiota across different lactation stages
Tapia Gonzalez [62]	2023	Mexico	Observational study	82	82 in 24–48 h pp.	To investigate whether the consumption of non-nutritive sweeteners during pregnancy could be related to changes in the colostrum microbiota
Wang [67]	2023	China	Observational study	15	3 in 1 d pp, 3 in 14 d pp, 3 in 20 d pp, 3 in 30 d pp, 3 in 90 d pp	To investigate the microbiological diversity and the correlation between breastmilk and infant gut
Du [41]	2022	China	Observational study	31	31 (colostrum)	To verify the entero-mammary pathway of breast milk by investigating the microbiota of colostrum and nipple skin in mothers who were separated from their newborns at birth
Ji [46]	2022	China	Observational Study	25	25 pp	To investigate the short- and long-term effects of perinatal antibiotic treatments on breast milk and infant gut microbiota, including the transfer of antibiotic resistance genes
Karampatsas [47]	2022	Gambia	Prospective observational study	107	32 right after birth, 9 in 60 d	To characterize the changes in breast milk microbiota and metabolomic profiles during the first 60 days of lactation and their relationship to infant gut and respiratory microbiota development
Li [49]	2022	China	Prospective observational study	23	6 right after birth, 19 in 30 d pp	To compare the influence of breast milk microbiota on the development and colonization of infant gut microbiota and short-chain fatty acids in exclusively breastfed vs. mixed-fed healthy infants
Liu [50]	2022	China	Longitudinal observational study	53	39 in 3–5 d pp, 44 in 13–15 d pp, 51 in 1 mo pp, 39 in 4 mo pp, 31 in 6 mo pp	To investigate the diversity, temporal dynamics, and influencing factors of the breast milk microbiome over the first six months postpartum in healthy Chinese breastfeeding women
Qi [55]	2022	China	Longitudinal observational study	19	19 in 0–7 d pp, 19 in 1–14 d pp, 19 in >42 d pp	To investigate the transmission of maternal gut-associated bacteria to the infant’s gut via breast milk at different lactation stages
Wang [66]	2022	China	Observational study	30	30 within 30 h pp	To investigate the effect of maternal antibiotic exposure on the microbiota of the colostrum
Xie [71]	2022	China	Observational study	97	97 in 2–5 d pp	How ethnicity affects maternal milk microbiota
Gámez-Valdez [44]	2021	Mexico	Cross-sectional observational study	43	43 within 24 h pp	To characterize the impact of maternal gestational diabetes mellitus and obesity on breastmilk microbiota composition
Nikolopoulou [53]	2021	Greece	Cross-sectional observational study	100	26 (colostrum), 74 (mature milk)	To detect *Lactobacillus* and *Bifidobacterium* spp. in colostrum and mature breast milk and to investigate the influence of demographic and nutritional factors on their presence
Corona-Cervantes [35]	2020	Mexico	Descriptive study	67	67 in 1–6 d pp	To evaluate the association of human milk bacteria and the delivery mode with the neonate gut bacterial composition
Tao [61]	2020	China	Observational study	104	104 (colostrum), 86 (not specified)	To compare the colostrum microbiota from healthy breastfeeding women and the milk from mastitis patients
Wan [65]	2020	China	Longitudinal observational study	117	117 in 1 d pp, 113 in 14 d pp, 104 in 6 wks pp	To investigate the human milk microbiota during lactation and examine the associations of maternal geographic location, diet, age, and gestational hypertensive status with milk microbiota
Cabrera-Rubio [32]	2019	Spain		25	25 in <5 d pp, 25 in <15 d pp, 25 in 1 mo pp	To investigate the impact of the FUT2 genotype on the milk microbiota during the first month of lactation and the association with HMO
Tang [60]	2019	China	Cross-sectional observational study	89	29 in 5 d pp	The aim of the study was to investigate the association between environmental persistent pollutants and the microbial composition of human colostrum
Togo [17]	2019	France	Observational study	128	118 in 2 d pp, 20 in 10 d pp	The aim of this study was to investigate the presence of living methanogenic archaeain in human colostrum and mature milk
Williams [69]	2019	Russia	Prospective longitudinal observational study	21	19 in 2 d pp, 14 in 5 d pp, 17 in 1 mo pp, 15 in 2 mo pp, 14 in 3 mo pp, 10 in 4 mo pp, 10 in 5 mo pp, 12 in 6 mo pp	To investigate whether human milk microbiomes are correlated with those of oral and fecal samples of healthy lactating women and their infants
Chen [33]	2018	Taiwan	Observational study	33	20 in 5 d pp, 10 in 6–15 d pp, 3 in >15 d pp	To investigate whether milk from healthy mothers harbors potential probiotics
Tuominen [64]	2018	Finland	Observational study	31	31 (colostrum), 4 in 2mo pp	To investigate the association between microbiota in breast milk and the infant mouth
Aakko [28]	2017	Finland	Observational study	11	11 within 24 h pp	To investigate whether the composition of human milk oligosaccharides in colostrum influences the microbial composition of the milk
Boix-Amorós [30]	2017	Spain	Pilot study	41	16 in 1–6 d pp, 14 in 7–14 d pp, 28 in >/=15 d pp	To investigate the presence, load, composition, and potential viability of fungal organisms in human breast milk from healthy lactating mothers, and to explore their association with milk components such as macronutrients and somatic cells
Damaceno [36]	2017	Brazil	Observational study	47	47 within 30 min pp, 47 in 5–9 d pp, 47 in 25–30 d pp	To identify potential probiotic bacteria in human milk and assess their association with maternal factors
Dewanto [39]	2017	Indonesia	Randomized clinical trial	75	70 in 1–5 d pp, 70 in 3 mo pp	To evaluate whether supplementation with the probiotic *Bifidobacterium animalis* subsp. lactis HNO19 from the third trimester of pregnancy affects probiotic presence in breast milk and markers of infant gut mucosal integrity at birth and three months postpartum
Toscano [63]	2017	Italy	Observational study	29	29 in 3 d pp	To assess the impact of delivery mode on the microbiota of colostrum
Williams [68]	2017	Russia	Observational study	21	5 in 2 d pp, 12 in 5 d pp, 9 in 10 d pp, 17 in 1 mo pp, 15 in 2 mo pp, 14 in 3 mo pp, 10 in 4 mo pp, 10 in 5 mo pp, 12 in 6 mo	To describe the human milk microbiome and assess the correlations with maternal nutrient intake, time postpartum, delivery mode, and body mass index
Boix-Amorós [29]	2016	Spain	Observational study	21	56	To investigate the relationships between breast milk microbiota composition, bacterial load, macronutrients, and human cells during lactation in samples from healthy mothers over time
Dave [37]	2016	USA	Pilot study	10	9 in 2–4 d pp	To explore associations between the breast milk microbiome and the salivary microbiome of 5-year-old children
Drago [40]	2016	Italy	Observational study	50	50 in 3 d pp, 32 in 1 mo pp	To analyze and compare the microbiota networks in colostrum and mature milk to reveal dynamic bacterial interactions and population differences
Sakwinska [56]	2016	China	Cross-sectional observational study	90	30 in 0–4 d pp, 30 in 5–11 d pp, 30 in 1–2 mo pp	To examine the microbiota of breast milk at different lactation stages and assess the impact of collection method, delivery mode, and lactation stage
Mastromarino [51]	2015	Italy	Double blind randomized controlled trial	66	66 right after birth, 66 in 1 mo pp	To evaluate the effect of oral probiotic supplementation with VSL#3 during late pregnancy and lactation on breast milk microbiota and functional components
Moles [52]	2015	Spain	Observational study	22	17 (colostrum), 34 (mature milk)	To investigate how extremely premature birth (24–27 weeks gestation) affects the microbiological, biochemical, and immunological composition of colostrum and mature milk
Khodayar-Pardo [48]	2014	Spain	Observational Study	32	32 in 1–5 d pp, 32 in 6–15 d pp, 32 in >16 d pp	To analyze how lactation stage, gestational age, and delivery mode influence breast milk microbiota composition
Obermajer [54]	2014	Slovenia	Cross-sectional observational study	45	45 in 2–3 d pp	To characterize the microbial community composition and prevalence of bacteriocin genes in colostrum samples from 45 healthy Slovenian mothers
Cabrera-Rubio [31]	2012	Finland	Observational study	18	18 in 0–2 d pp, 18 in 1 mo pp, 18 in 6 mo pp	To identify pre- and postnatal factors influencing human milk microbiome
Collado [34]	2012	Finland	Observational study	56	43 within 24–48 h pp, 44 in 1 mo pp, 34 in 6 mo pp	To analyze the relationship between cytokines and microbiota and to explore the maternal influences on these
Dubos [42]	2011	Chile	Observational study	116	116 within 2 d pp	To evaluate the biodiversity of *Lactobacillus* spp. in breast milk from Chilean mothers and assess the resistance of isolated strains to gastric pH and bile salts as potential probiotics.
Solis [58]	2010	China	Longitudinal observational study	20	20 in 1 d pp, 20 in 10 d pp, 20 in 30 d pp, 20 in 90 d pp	To evaluate the establishment of lactic acid bacteria (LAB) and *bifidobacteria* in the gut microbiota of 20 vaginally delivered, breastfed full-term infants over the first 3 months of life
Martin [72]	2003	Spain	Cross-sectional observational study	8	16 in 4 d pp	To investigate the presence and potential probiotic role of lactic acid bacteria in human breast milk
Wyatt [70]	1969	Guatemala	Observational study	31	51 in 2 d pp	To investigate the human milk microbiome of women in women of low socio-economic groups in Guatemala

N; number, min; minutes, h; hours, wks; weeks, mo; months, d; days, pp; postpartum.

**Table 2:** Results of the included studies.

First Author	Milk Sampling Method	Analysis Method	Microorganisms	Main Bacterial Phyla, Genera, Species	Main Findings/Outcomes
Deflorin [39]	Breast pump	16S rRNA	N/A	*Staphylococcaceae*, *Streptococcaceae*, *Moraxellaceae*, *Pseudomonadaceae*, *Lactobacillaceae*, *Gemellaceae*, *Micrococcaceae*, *Burkholderiaceae*, *Xanthomonadaceae*	Dominant Genera: *Staphylococcus* (42.30%), *Streptococcus* (38.55%), Gemella (6.40%) No significant associations between mental health and alpha or beta diversity (*p* > 0.05) After FDR correction: Prenatal GAS negatively correlated with the following: Class: *Alphaproteobacteria* (τ = −0.20, FDR = 0.05) Order: *Pseudomonadales* (τ = −0.20, FDR = 0.08) Postpartum STAI-S negatively correlated with the following: Orders: *Propionibacteriales*, *Pseudomonadales*, *Caulobacterales* Genera: *Cutibacterium*, *Pseudomonas*_N, AUCg at T1 negatively correlated with genus *Stenotrophomonas* (τ = −0.24, FDR = 0.05) No correlations between total cortisol decline and any microbial taxa (*p* > 0.05) No associations with alpha or beta diversity (*p* > 0.05), HM cortisol negatively correlated with the following: Family *Gemellaceae* (τ = −0.24, FDR = 0.06) Genus *Gemella* (τ = −0.24, FDR = 0.03) Species *Gemella haemolysans* (τ = −0.24, FDR = 0.06) Species *Streptococcus mitis* (τ = −0.24, FDR = 0.03) No associations between the following: HM cortisol and alpha/beta diversity (FDR > 1) HM cortisone and microbial diversity or taxa (FDR > 0.1, *p* > 0.05)
Ge [45]	N/A	16S rRNA	N/A	*Proteobacteria*, *Firmicutes*, *Actinobacteriota*, *Bacteroidota*, *Deinococcota Cyanobacteria*, *Acidobacteriota*, *Chloroflexi*, *Patescibacteria*	Higher microbial abundance in colostrum than nipple skin (Chao1 and Simpson indices, *p* < 0.05). Beta diversity (unweighted UniFrac) showed distinct microbial structures (*p* < 0.01, PERMANOVA). 170 OTUs were shared, but 111 were unique to colostrum. Infant Factors Gender: No significant effect on alpha or beta diversity Female Newborns: Higher abundance of *Streptococcaceae* (LDA > 3.0) Male Newborns: Higher abundance of *Roseburia* and *Alcaligenaceae* (LDA > 3.0), *Roseburia* verified by edgeR Gestational Age: No significant effect on diversity Full-Term Births: Higher abundance of *Bifidobacteria* (LEfSe and edgeR verified) Maternal Factors Parity: No effect on alpha or beta diversity Delivery Mode: No significant diversity changes Cesarean Section: Higher abundance of *Bifidobacterium* (LDA > 3.0, verified by edgeR) Intrapartum Antibiotic Prophylaxis (IAP): No effect on IAP Group: Higher abundance of *Lachnospiraceae* (LDA > 3.0, verified by edgeR) Non-IAP Group: Higher abundance of *Lactobacillus* (LDA > 3.0, verified by edgeR) Type of Antibiotic (cefazolin vs. cefuroxime): No significant difference in diversity
Sun [59]	Manually	16SrRNA gene quantification, PCR.	N/A	N/A	Associations Between Bacteria and Human Milk Oligosaccharides (HMOs): *Lactobacillus* showed a positive correlation with LNT (r = 0.250, *p* = 0.037). *Staphylococcus* showed a negative correlation with DS-LNT (r = −0.240, *p* = 0.045). *Streptococcus* showed positive correlations with the following: LNFPII (r = 0.314, *p* = 0.011) LNFP III (r = 0.251, *p* = 0.044) 3′-Sialyllactose (3-SL) (r = 0.322, *p* = 0.009) LNnT (r = 0.292, *p* = 0.018)
Fernández-Tuñas [43]	Breast pump	16S rRNA	N/A	*Proteobacteria*, *Firmicutes*	Maternal Stress: High stress was associated with lower microbial diversity (Shannon index) at all time points. Stress affected microbiome composition, leading to higher levels of *Proteobacteria* and lower *Firmicutes* over time. In mothers with low stress, *Firmicutes* remained dominant, and changes in *Proteobacteria* were less pronounced.
Singh [57]	Manually	Gene sequencing 16S rRNA	Amplicon Sequence Variants in colostrum: 339	*Firmicutes*, *Parcubacteria*, *Actinobacteria*, *Proteobacteria*, *Bacteroidetes*, *Streptococcus*, *Staphylococcus* *Veillonella*, *Corynebacterium*, *Propionibacterium*, *Lactobacillus*	Microbial Diversity Assessment: Alpha Diversity: Colostrum had the lowest diversity, which increased in transitional and mature milk. Significant differences were observed across lactation stages (padj = 0.006) using Observed OTUs and Shannon’s Index. Beta Diversity: Significant stage-specific differences were identified: Weighted UniFrac: *p* = 0.012 Jaccard Distance: *p* = 0.001 Comparison: Preterm vs. Full-term Milk Preterm Milk: Higher enrichment and diversity (padj < 0.0001). Enriched phyla: *Actinobacteria*, *Bacteroidetes*. Dominant genera: *Faecalibacterium*, *Prevotella*, *Clostridium*, *Bacteroides*, *Enterobacter*. Dominant species: *Staphylococcus haemolyticus*, *Propionibacterium acnes*. Full-term Milk: Enriched phylum: OD1 (padj < 0.0001). Dominant species: *Staphylococcus epidermidis*, unclassified OD1, unclassified *Veillonella*. Effect of Antibiotics: No significant impact on the composition or diversity of the breast milk microbiome.
Tapia Gonzalez [62]	Manually	16S rRNA gene sequencing	N/A	*Proteobacteria*, *Bacteroidota*	Associations with Artificial Sweetener Consumption (NNS): *Bifidobacterium*: Positive association with higher NNS consumption (no statistical significance). *Prevotella*: Positive association with increased NNS consumption (no statistical difference). *Staphylococcus* and *Blautia*: Both decreased with higher NNS consumption. *Streptococcus*: Higher levels in Q4 compared to Q1 and Q2. Maternal DNA: Lower levels in Q3 and Q4, indicating a decrease in bacterial abundance with higher NNS consumption.
Wang [67]	Breast pump	16SrRNA gene sequencing, qPCR	Total 16s rRNA: 2,520,825 16SrRNA	*Proteobacteria*, *Firmicutes*, *Actinobacteria*, *Bacteroidetes*	Common Bacteria Between Maternal Milk and Infant Feces: *Streptococcus*, *Bacteroides*, and *Lactobacillus* were found in both maternal milk and infant feces, suggesting bacterial transfer from mother to infant. *Acinetobacter* was detected in both locations, indicating vertical transfer of microbiome. Changes Over Time: *Acinetobacter* and *Stenotrophomonas* were abundant in colostrum but decreased over time. *Bacteroides* and *Lactobacillus* remained common in both milk and feces throughout the study period. Diversity and Abundance: Shannon Index: Higher microbial diversity in colostrum, which decreased in subsequent stages of lactation. Chao Index: The highest bacterial abundance was observed in colostrum and decreased over time.
Du [41]	Manually	qPCR, gene sequencing, 16S rRNA	OTUs: 11,190	*Firmicutes*, *Proteobacteria*, *Bacteroidetes*, *Actinobacteria*, *Streptococcus*, *Staphylococcus*, *Enterococcus*	Mode of Delivery (Vaginal vs. Cesarean Section): No significant effect on alpha and beta diversity. LEfSe Analysis: Cesarean section: Enriched in *Bifidobacterium* (LDA score > 3.0). Perinatal Antibiotic Prophylaxis (IAP): No significant effect on alpha and beta diversity. LEfSe Analysis: With IAP: Enriched in *Lachnospiraceae* (LDA score > 3.0). Without IAP: Enriched in *Lactobacillus* (LDA score > 3.0). Infant Gender (Boy vs. Girl): No significant effect on alpha and beta diversity. LEfSe Analysis: Girls: Enriched in *Streptococcaceae* (LDA score > 3.0). Boys: Enriched in *Roseburia* and *Alcaligenaceae* (LDA score > 3.0). Observations Regarding Parity: No significant effect on alpha and beta diversity. Gestational Age (Term vs. Preterm Birth): No significant effect on alpha and beta diversity. LEfSe Analysis: Term Infants: Enriched in *Bifidobacteria* (LDA score > 3.0).
Ji [46]	N/A	16S rRNA	OTUs: Treatment with cefuroxime: 18 OTUs increased, 3 OTUs decreased. Treatment with cefuroxime + cefixime: 8 OTUs increased, 5 OTUs decreased.	*Firmicutes*, *Actinobacteria* *Streptococcus*, *Staphylococcus*, *Rothia*	Microbial Richness and Diversity: No significant differences in alpha diversity indices were found between the groups receiving CXM, CXM + CFX, and the control group. PCoA Analysis: The analysis based on the unweighted UniFrac distance did not show significant differences in the composition of the microbial community between the groups (*p* > 0.05, Adonis analysis). Dominant Bacteria: No significant dominance at the level of phylum or genus was observed in the groups receiving CXM or CXM + CFX compared to the control groups (Kruskal–Wallis rank-sum test).
Karampatsas [47]	Manually	16S rRNA	N/A	*Streptococcus*, *Staphylococcus*, *Gemella*.	Changes in Microbial Diversity and Composition: Colostrum and mature milk exhibited distinct microbial profiles, reflecting changes in diversity and specific genera during the lactation period.
Li [49]	Manually	16S rRNA	N/A	*Firmicutes*, *Actinobacteriota*, *Proteobacteria*, *Streptococcus*, *Acinetobacter*, *Pseudomonas*, *Brevundimonas*, *Serratia*. *Veillonella*, *Escherichia-Shigella*, *Bacillus*, *Rothia*, *Gemella*, *Corynebacterium*, *Ammoniphilus*, *Clostridium*, *Listeria*, *Erysipelatoclostridium*, *Citrobacter*	Comparison of Exclusive Breastfeeding (BF) and Mixed Feeding (MF): No significant differences in the microbiome of maternal milk between BF and MF groups after adjusting for feeding type. Correlations between Maternal Milk Microbiome and Infant Gut Microbiome (Day 0, BF Group): Positive Correlations: *Lactobacillus* (in milk) with *Bifidobacterium* (r = 1.000, *p* < 0.001) and *Clostridium* (r = 0.900, *p* = 0.037) in infant gut. *Enterobacter* (in milk) with *Lactobacillus* (r = 0.900, *p* = 0.037) in infant gut. Negative Correlations: *Klebsiella* (in milk) with *Lactobacillus* (r = −1.000, *p* < 0.001) in infant gut. *Escherichia-Shigella* (in milk) with *Ammoniphilus* (r = 0.900, *p* = 0.037) in infant gut. Correlations with Short-Chain Fatty Acids (SCFAs) (Day 30): BF Group: Negative correlation with acetic acid (r = −0.900, *p* ≤ 0.037) for *Desulfobacterota*, *Bacteroidota*, *Proteobacteria*. MF Group: Positive Correlations with SCFAs: *Ammoniphilus*, *Haemophilus* (acetic acid). *Rothia* (propionic acid, r ≥ 0.600, *p* ≤ 0.037). Microbial Diversity by Feeding Type: BF Group: More consistent and specific correlations between maternal milk and infant gut microbiome. MF Group: Greater diversity in associated bacteria, reflecting the additional microbiota introduced through mixed feeding.
Liu [50]	Breast pump	16S rRNA	N/A	*Proteobacteria*, *Firmicutes*, *Bacteroidetes*, *Actinobacteria*, *Staphylococcus*, *Gemella*, *Streptococcus*, *Acinetobacter*.	Alpha Diversity: High Shannon diversity index in colostrum compared to later stages of lactation, indicating greater microbial diversity at this early stage. Beta Diversity: Significant differences in microbial composition between colostrum and subsequent stages of lactation, reflecting distinct microbiome profiles at different time points. Cluster Analysis: Colostrum samples predominantly grouped in Cluster 2, characterized by high levels of *Streptococcus* and *Staphylococcus*.
Qi [55]	N/A	Gene sequencing 16S rRNA, shotgun sequencing	Total Amplicon Sequence Variants in infant microbiome from colostrum to mature milk: 4328 ASVs	*Bifidobacterium*, *Fusicatenibacter*, *Blautia*, *Staphylococcus*, *Bifidobacterium*, *Streptococcus salivarius*	Observations on Microbial Diversity: Higher microbial diversity was observed in colostrum compared to mature milk. Significant differences in alpha diversity were found between maternal feces and other sample types (*p* < 0.01). Beta diversity analysis revealed significant shifts between breast milk and neonatal microbiota across all stages of lactation (*p* < 0.05). Specific Bacterial Transfer: *Streptococcus salivarius*: Transferred during all lactation stages except transitional milk. *Bifidobacterium longum*: In <30% of Mother–Infant pairs: Transferred from maternal gut to infant gut. In 30% of Pairs: Transferred from breast milk to infant gut during the mature milk stage. Escherichia coli: Transfer began in the transitional milk stage and continued into mature milk. Functional Metabolic Pathways: Distinct pathways were identified in infant feces: Total of 9 pathways associated with colostrum. Total of 16 pathways associated with mature milk.
Wang [66]	Manually	16S rRNA gene sequencing	Bacterial cells: 10^3^–10^6^/mL	*Firmicutes*, *Proteobacteria*	Diversity (Alpha Diversity): Shannon Index: AT Group: 3.02 NT Group: 3.44 Significant difference with *p* = 0.026 Community Differentiation (Beta Diversity): Bray–Curtis Distance: Significant differences between the AT and NT groups. PERMANOVA *p* = 0.001 Microbial Ecological Network: AT Group: More complex network with 167 nodes and 581 connections. NT Group: Fewer connections with 361 connections.
Xie [71]	Manually	16S rRNA gene sequencing	Amplicon Sequence Variants: 789 ASVs in 97 samples	*Proteobacteria*, *Firmicutes*	Nutritional Associations: Energy Intake: Positive correlation with *Gemella* (rs = 0.58, *p* = 0.006). Macronutrients: Saturated and Monounsaturated Fatty Acids: Negative correlation with *Corynebacterium* (rs = −0.59, *p* = 0.005, and rs = −0.46, *p* = 0.036, respectively). Carbohydrates: Negative correlation with *Firmicutes* (rs = −0.54, *p* = 0.011; rs = −0.47, *p* = 0.031; rs = −0.51, *p* = 0.018, respectively). Micronutrients: Pantothenic Acid: Negative correlation with *Streptococcus* (rs = −0.44, *p* = 0.043). Riboflavin and Calcium: Positive correlation with *Veillonella* (rs = 0.52, *p* = 0.016; rs = 0.58, *p* = 0.006, respectively). Thiamine, Niacin, Folic Acid, Vitamin B-6, Chromium: Negative correlation with *Lactobacillus* (rs = −0.51, *p* = 0.005; rs = −0.51, *p* = 0.005; rs = −0.54, *p* = 0.003; rs = −0.48, *p* = 0.01; rs = −0.49, *p* = 0.009, respectively). Ethnicity Associations: Microbiome richness: Han mothers had higher microbial diversity (Chao1: 151.54) compared to Li mothers (106.75, *p* = 0.001). Gender differences: Li group: *Proteobacteria* (66.5%) > *Firmicutes* (29.5%). Han group: *Firmicutes* (46.5%) > *Proteobacteria* (43.7%). Genus-level Differences: Li Group: Cupriavidus (26.28%), *Staphylococcus* (17.36%), *Streptococcus* (13.11%). Han Group: *Acinetobacter* (28.72%), *Staphylococcus* (28.38%), *Streptococcus* (9.45%). *Lactobacillaceae* and *Bifidobacterium*: Han Group: *Bifidobacterium* detected in 60% of samples; *Limosilactobacillus reuteri* detected in 11.67% of samples. Li Group: Neither Limosilactobacillus reuteri nor Bifidobacterium were detected.
Gámez-Valdez [44]	Manually	16S rRNA	N/A	*Staphylococcus*, *Prevotella*, *Corynebacterium 1*, *Anaerococcus*, *Burkholderia*, *Rhodobacteraceae*, *Xanthobacteraceae*	Women with Obesity (Ob-F): Higher levels of *Gemella* and *Staphylococcus* (*p* < 0.10). Women with GDM (GD-F): Higher levels of *Prevotella* and *Rhodobacteraceae* compared to GD-M and controls (*p* < 0.10). Summary of Comparisons: *Staphylococcus* and *Anaerococcus*: Elevated in the obesity and GDM groups (*p* < 0.05). *Prevotella*: Significantly higher in GDM compared to controls (*p* < 0.10) and obesity (*p* < 0.05). Alpha Diversity: Lower in obese men (*p* < 0.05). Higher in colostrum from women with GDM compared to controls (*p* < 0.05).
Nikolopoulou [53]	Breast pump	Real-time PCR	N/A	*Bifidobacterium*, *Lactobacillus*	Probiotic Supplement Intake: 60% of samples were positive in women who took probiotic supplements. Total of 53.3% of samples were positive in women who did not take probiotics (no significant difference). Yogurt Consumption: 64.3% of samples were positive in women who consumed yogurt; significantly higher compared to those who did not consume it.
Corona-Cervantes [35]	manually	16S rRNA	Proteobacteria cells: 55.40% ± 32.1	*Proteobacteria*, *Firmicutes*, *Actinobacteria*, *Bacteroidetes*	Lactation Stage: Higher bacterial count in colostrum compared to later milk samples (*p* = 0.00001). Delivery Mode and BMI: *Lactobacillus gasseri:* Isolated exclusively in vaginal deliveries with normal BMI. *Bifidobacterium breve:* Found in one sample from a vaginal delivery with normal BMI. *Streptococcus salivarius*: Detected in samples from cesarean deliveries. Maternal Age: A slight decrease in bacterial richness (Chao1) with increasing age. Days After Delivery: Shannon diversity tends to increase over time after delivery.
Tao [61]	Manually	qPCR, real-time PCR, cultures	N/A	*Firmicutes* *Actinobacteria* *Proteobacteria* *Bacteroidetes* *Lactobacillus* *Bifidobacterium* *Staphylococcus* *Streptococcus*	Comparison of Colostrum and Milk from Mastitis: Higher abundance of *Lactobacillus*, *Bifidobacterium*, *Staphylococcus*, and *Streptococcus* (*p* < 0.01) in colostrum from healthy women compared to milk from mastitis. *Bifidobacterium* levels in colostrum were higher than in both the infected and non-infected breast tissue of patients with *Staphylococcus*-associated mastitis (*p* < 0.01). *Lactobacillus* and *Bifidobacterium* levels were significantly higher than *Staphylococcus* and *Streptococcus* (*p* < 0.0001). No significant differences in the quantity of bacteria between the two breasts in healthy women.
Wan [65]	Manually	16SrRNA gene sequencing, qPCR	N/A	*Firmicutes*, *Proteobacteria*, *Staphylococcus*, *Streptococcus*, *Lactobacillus*, *Acinetobacter*, *Pseudomonas*	Geographical Location: Significant differences in microbial diversity and the number of species (pforinteraction < 0.001 for diversity, *p* = 0.02 for the number of species). The microbial composition at the genus level differed significantly between three cities (PERMANOVA *p* < 0.001). Gestational Hypertension: Lower microbial diversity and number of species in the colostrum of mothers with gestational hypertension compared to healthy mothers (*p* < 0.05). Decreased Abundance of *Lactobacillus* in the Colostrum of Mothers with Prehypertension: *p* = 0.09 (colostrum) *p* = 0.004 (transitional milk) Mother’s Age: No significant differences in microbial diversity or the number of species between mothers aged ≤ 30 and >30: pforinteraction = 0.35 (diversity) *p* = 0.79 (number of species) Dietary Influence on Colostrum Microbiome: No significant association between maternal dietary factors and the composition of the colostrum microbiome at the genus level (*p*-values > 0.05 after FDR correction).
Cabrera-Rubio 2019 [32]	Breast pump	qPCR, 16S rRNA, Pyrosequencing	N/A	*Lactobacillus* spp., *Streptococcus* spp., *Enterococcus* spp. *Bifidobacterium* spp.	*Lactobacillus*: Positive correlation with 2′FL (ρ = 0.542, *p* = 0.038). *Staphylococcus*: Lower levels are associated with lower total and neutral HMO concentrations. *Streptococcus*: No correlation with HMO levels in colostrum, but a correlation is observed in transitional milk.
Tang [60]	N/A	16SrRNA gene sequencing, qPCR	N/A	*Proteobacteria*, *Firmicutes*, *Pseudomonas*, *Acinetobacter*, *Stenotrophomonas*, *Delftia*, *Enterococcus*	Effect of Exposure to γ-Hexachlorocyclohexane (g-HCH): *Pseudomonas*: Increased by 1.7 times in samples with high exposure to g-HCH. *Enterococcus*: Decreased by half in samples with high exposure to g-HCH. *Stenotrophomonas* and *Acinetobacter*: Increased in colostrum samples with high exposure to g-HCH.
Togo [17]	Manually	Culture, PCR, real-time PCR, 16SrRNA gene sequencing	Methanobrevibacter smithii. DNA: In colostrum: 463/mL In milk: 339/mL	*Methanobrevibacter smithii*, *Methanobrevibacter oralis*	Association with Maternal BMI: Obese Mothers: Lower frequency of M. smithii (14%). Non-obese Mothers: Higher frequency of M. smithii (33%). Overweight Mothers: Higher frequency of M. smithii (45%). Thin Mothers: 28% frequency of M. smithii. Associations with BMI: The BMI distribution before pregnancy was normal for mothers who were positive for M. smithii and skewed for those who were negative.
Williams 2019 [69]	Breast pump	16S rRNA gene sequencing, high-throughput sequencing	Total 16srRNA: 18,534,383	*Firmicutes*, *Bacteroidetes*, *Proteobacteria*, *Bacteroidetes*	Canonical Associations: Association Between Maternal Milk and Infant’s Oral Microbiome: Canonical correlation: 0.95 to 0.70 The first axis explains approximately 31% of the variation in the data. Association Between Maternal Milk and Infant’s Feces: Canonical Correlation: 0.80 The first axis explains approximately 29% of the variation. Association Between Maternal Milk and Maternal Feces: Canonical correlation: 0.72 (*p* = 0.0083) Microbiome Source Distribution in Maternal Milk: Contribution of Infant’s Oral Microbiome: Day 2: ~21% Month 5: ~66% Contribution of Mother’s Oral Microbiome: Day 2: ~26% Month 1–6 postpartum: 2–6%
Chen [33]	Manually	16S rRNA, qPCR	Bacteria: 154 species	*Staphylococcus*, *Streptococcus*, *Rothia*, *Enhydrobacter*	Cluster Analysis: Nine colostrum samples were grouped together, indicating consistent bacterial patterns among mothers. Changes in Bacterial Composition: Higher levels of *Enhydrobacter* and *Staphylococcus* in colostrum compared to transitional milk. Delivery Mode and Presence of Lactobacilli: Vaginal Delivery: 30% of the samples (4/13). Cesarean Section: 15% of the samples (3/20).
Tuominen [64]	N/A	16S rRNA gene sequencing, qPCR	N/A	*Firmicutes*, *Proteobacteria*, *Actinobacteria*, *Bacteroides* *Streptococcus*, *Staphylococcus*, *Gemellaceae*, *Rothia*, *Veillonella*, *Haemophilus*, *Propionibacterium*, *Corynebacterium* *Prevotella*, *Pseudomonas*, *Veillonelladispar*, *Bifidobacterium*, *Methanobrevibacter*	HPV and Microbiome Composition: HPV-positive samples exhibited a different microbial profile compared to HPV-negative samples (*p* = 0.036, RDA). *Veillonelladispar* was significantly higher in HPV-negative samples, both at the genus level (*p* = 0.025) and species level (*p* = 0.048). Higher microbial diversity was observed in breast milk compared to the infant’s oral cavity (*p* = 0.0043, Shannon index). There were no significant differences in the number of bacterial species (*p* = 0.394, Chao1 index). Differences Between Modes of Delivery: Colostrum from cesarean section contained more environmental microorganisms (e.g., *Pseudomonas*, *Enterococcus*) compared to vaginal delivery samples. Colostrum from vaginal delivery had significantly higher levels of *Streptococcus* (49% higher) and *Haemophilus* (94% higher) compared to colostrum from cesarean section.
Aakko [28]	manually	qPCR	Bacterial cells 10^5.1^/g	*Bifidobacterium* spp. *Bifidobacterium longum group* *Bifidobacterium breve* *Staphylococcus* spp. *Staphylococcus aureus* *Streptococcus group* *Lactobacillus group* *Akkermansia muciniphila* *Bacteroides-Prevotella group* *Clostridium cluster XIVa-XIVb* *Clostridium cluster IV*	Total HMO Concentration: Positive correlation with *Bifidobacterium* spp. (ρ = 0.63, *p* = 0.036). Sialylated HMOs: Positive correlation with Bifidobacterium breve (ρ = 0.84, *p* = 0.001). Fucosylated HMOs: Positive correlation with *Akkermansia muciniphila* (ρ = 0.70, *p* = 0.017). Non-fucosylated, Non-sialylated HMOs: Positive correlation with *Bifidobacterium longum* group (ρ = 0.65, *p* = 0.030). Fucosylated and Sialylated HMOs: Positive correlation with *Staphylococcus aureus* (ρ = 0.75, *p* = 0.007).
Boix-Amorós 2017 [30]	manually	qPCR, Pyrosequencing, cultures	Fungal load: 4.1 × 10^5^/mL	*Malassezia* *Candida* *Saccharomyces* *Lodderomyces*	Detection of Fungal DNA in 89% of Colostrum Samples. *Malassezia*: Positive correlation with bacterial load (ρ = 0.93, *p* = 0.007) and lactose content (ρ = 0.78, *p* = 0.048). Fungal Load: Positive correlation with fat content and non-fat solid content of the milk.
Damaceno [36]	Manually	Cultures, MALDI-TOFMS, PCR	Bacterial cells: 3.9 log10 CFU/mL (95% CI: 3.57–4.22)	*Staphylococcus*, *Streptococcus salivarius*, *Bifidobacterium breve*, *Lactobacillus gasseri:*	Delivery Mode and BMI *Lactobacillus gasseri*: Isolated exclusively in vaginal deliveries with normal BMI. *Bifidobacterium breve:* Found in only one sample from a vaginal delivery with normal BMI. *Streptococcus salivarius*: Detected in samples from cesarean deliveries. Lactation Stage: The total bacterial count was significantly higher in colostrum compared to later milk samples (*p* = 0.00001).
Dewanto [39]	Manually	Real-time PCR	At Delivery (V0): Probiotic Group: Median 963.8 copies/mL Placebo Group: Median 2523.2 copies/mL (*p* = 0.242) At 3 Months (V3): Probiotic Group: Median 1803.6 copies/mL Placebo Group: Median 2201.1 copies/mL (*p* = 0.819)	*Bifidobacterium animalis lactis*, *Lactobacillus*	Presence of DR10 in Colostrum: Total of 14% of mothers in the probiotic group had detectable DR10 levels, compared to 0% in the placebo group. DR10 was exclusively transferred to colostrum in the probiotic group. IL-8 Levels in Colostrum: Probiotic Group: Median 2810.1 pg/mL (range: 94.5–66,246.9 pg/mL). Placebo Group: Median 1516.4 pg/mL (range: 28.8–514,157 pg/mL). No significant difference between the groups (*p* = 0.327).
Toscano [63]	Manually	16S rRNA gene sequencing	N/A	*Streptococcus*, *Haemophilus*, *Achromobacter*, *Rhodanobacter*, *Faecalibacterium*, *Clostridium*, *Staphylococcus*, *Finegoldia*, *Halomonas*, *Prevotella*, *Pseudomonas*, *Ruminococcus*, *Peptostreptococcus*, *Roseburia*, *Serratia*, *Akkermansia*.	General Findings: Anaerobic Bacteria: Both types of colostrum (vaginal delivery and cesarean section) were dominated by anaerobic genera, which made up approximately 65% of the detected microbiome. Bacterial Hubs: *Achromobacter* and *Staphylococcus* were central in both delivery modes, while *Rhodanobacter*, *Ruminococcus*, and *Serratia* showed similar roles as hubs in both cases. Environmental Bacteria: Colostrum from cesarean section contained more environmental microorganisms, suggesting a potential influence of the hospital environment. Higher Microbial Diversity: Colostrum from vaginal delivery exhibited slightly higher biodiversity, with increased Shannon and Chao indices compared to colostrum from cesarean section, although the differences were not statistically significant.
Williams 2017 [68]	Breast pump	16S rRNA gene sequencing, real-time PCR	Bacterial cells: 10^3^–10^6^/mL	*Firmicutes*, *Actinobacteria*, *Proteobacteria*, *Bacteroidetes*	Maternal BMI (Body Mass Index): Overweight/Obese Women: Higher levels of *Granulicatella* (1.8% ± 0.6%) compared to women of normal weight (0.4% ± 0.2%, *p* < 0.05). Mode of Delivery: Cesarean Section: Higher levels of *Propionibacterium* (1.9% ± 0.7%) compared to vaginal delivery (0.8% ± 0.2%), *p* = 0.066. Infant’s Gender: Male Infants: Higher levels of *Streptococcus* (51.7% ± 4.2%) and lower levels of *Staphylococcus* (19.2% ± 3.7%) compared to female infants. Dietary Associations: Energy Intake: Positive association with *Gemella* (rs = 0.58, *p* = 0.006). Saturated Fatty Acids (SFA) and Monounsaturated Fatty Acids (MUFA): Negative association with *Corynebacterium* (rs = −0.59, *p* = 0.005 for SFA, rs = −0.46, *p* = 0.036 for MUFA). Carbohydrates: Negative association with *Firmicutes* (rs = −0.54, *p* = 0.011 for total carbohydrates, rs = −0.47, *p* = 0.031 for disaccharides, rs = −0.51, *p* = 0.018 for lactose). Pantothenic Acid: Negative association with *Streptococcus* (rs = −0.44, *p* = 0.043). Riboflavin and Calcium: Positive association with *Veillonella* (rs = 0.52, *p* = 0.016 for riboflavin, rs = 0.58, *p* = 0.006 for calcium). Thiamine, Niacin, Folate, Vitamin B-6, Chromium: Negative association with *Lactobacillus*: rs = −0.51, *p* = 0.005 (thiamine) rs = −0.51, *p* = 0.005 (niacin) rs = −0.54, *p* = 0.003 (folate) rs = −0.48, *p* = 0.01 (vitamin B-6) rs = −0.49, *p* = 0.009 (chromium) Microbial Changes Over Time: *Veillonella*: Increased significantly between the 4th and 6th month (*p* = 0.01). *Granulicatella*: Increased significantly between the 5th and 6th month (*p* = 0.01).
Boix-Amorós 2016 [29]	manually	16S RNA, qPCR	Bacterial cells: 10^6^/mL	*Staphylococcus* *Acinetobacter* *Staphylococcus*, *Acinetobacter*, *Finegoldia*, *Streptococcus*, *Corynebacterium*, *Peptoniphilus*, *Pseudomonas*.	Diversity: High bacterial species richness, with no significant differences compared to later stages of lactation. Distribution: 65.75% of the bacteria in colostrum were cell-associated, decreasing to 63.92% free bacteria in mature milk.
Dave [37]	Breast pump	16S rRNA, qPCR	OTUs: 241	*Streptococcus*, *Staphylococcus*, *Prevotella*, *Neisseria*	Comparison with the Child’s Saliva *Streptococcus*: No significant difference in abundance (73.8% in breast milk vs. 60.4% in child’s saliva). *Staphylococcus*: Present in breast milk (10.9%) but absent in the child’s saliva. Maternal BMI Before Pregnancy *Streptococcus*: Negative correlation with maternal BMI (r = −0.67, *p* = 0.048). Microbial Diversity: Positive correlation with maternal BMI (r = 0.77, *p* = 0.016).
Drago [40]	Manually	16S rRNA	OTUs: 4200	*Italy* *Abiotrophia* spp., *Actinomycetospora* spp., *Aerococcus* spp., *Alloiococcus* spp., *Amaricoccus* spp., *Bergeyella* spp., *Citrobacter* spp., *Desulfovibrio* spp., *Dolosigranulum* spp., *Faecalibacterium* spp., *Parasutterella* spp., *Rhodanobacter* spp., *Rubellimicrobium* spp. *Burundi:* *Aeribacillus* spp., *Agaricola* spp., *Alterythrobacter* spp., *Amaricoccus* spp., *Aquabacterium* spp., *Aquimonas* spp., *Brachybacterium* spp., *Dolosigranulum* spp., *Micrococcus* spp., *Peptostreptococcus* spp., *Propionibacterium* spp., *Serratia* spp.	Differences in Bacterial Abundance: Italy: Higher abundance of lactic acid bacteria in colostrum. Burundi: Higher prevalence of potential pathogens such as *Serratia* spp. and *Peptostreptococcus* spp. Influence of Diet and Geographical Location: Italy: A diet rich in animal proteins, fats, and sugars affected bacterial networks such as *Abiotrophia* spp. (colostrum) and *Parabacteroides* spp. (mature milk). Burundi: A diet based on plant fibers caused bacterial networks like *Aquabacterium* spp. (colostrum) and *Rhizobium* spp. (mature milk). Changes in Central Nodes (Central Node Shifts): Italy: From *Aciditerrimonas* spp. (colostrum) to *Alistipes* spp. (mature milk). Burundi: From *Sphingomonas* spp. (colostrum) to *Rhizobium* spp. (mature milk).
Sakwinska [56]	Breast pump	Gene sequencing 16S rRNA	Bacterial cells: Standard protocol: 7.5 × 10^4^ Aseptic protocol: 7.9 × 10^3^	*Staphylococcus*, *Streptococcus*, *Acinetobacter*	Microbiome Mapping (16S rRNA Gene Sequencing): Aseptic Protocol: Most samples were below detection limit (<10^6^ genome copies/mL). Total of 23 out of 30 samples yielded no detectable PCR product. Only 1 sample produced sufficient PCR amplicon. Standard Protocol: Showed higher bacterial DNA content. Total of 17 out of 60 samples yielded sufficient PCR product. Total of 10 samples had weak PCR product. Statistical Analysis: Significant differences were observed between the aseptic and standard protocols (*p* < 0.001, AMOVA). No differences were found related to lactation stage or mode of delivery.
Mastromarino [51]	Breast pump	Real-time PCR, 16S rRNA sequencing, spectroscopy NMR, ELISA	Colostrum Lactobacilli: Probiotic GroupQ: 4.5 × 10^3^ cells/mL Placebo Group: 6.6 × 10^2^ cells/mL Bifidobacteria: Probiotic Group: 1.7 × 10^4^ cells/mL Placebo Group: Lower numbers (no specific median value provided) Mature Milk (1 month after delivery): Lactobacilli: Probiotic Group: 5.8 × 10^3^ cells/mL Placebo Group: 9.8 × 10^2^ cells/mL Bifidobacteria: Probiotic Group: 1.4 × 10^4^ cells/mL Placebo Group: 3.1 × 10^3^ cells/mL	*Lactobacilli*, *Bifidobacteria*	Mode of Delivery: - Vaginal Delivery: - Higher levels of *Lactobacilli* and *Bifidobacteria* were observed in both colostrum and mature milk in the probiotic group compared to the placebo group. - Cesarean Section: - No significant differences were found between the probiotic group and the placebo group, likely due to the influence of antibiotics.
Moles [52]	Manually	MALDI-TOF MS	Bacterial load: Colostrum: 2.00–3.28 log_10_ CFU/mL Mature milk: 2.00–4.19 log_10_ CFU/mL	*Staphylococcus*, *Streptococcus*, *Lactobacillus*, *Enterococcus*, *Enterobacteria*.	Enterococci: Higher frequency in mature milk compared to colostrum (*p* = 0.000); *Lactobacilli*: More frequent in mature milk (*p* = 0.041); *Enterobacteria*: Increased presence in mature milk (*p* = 0.038).
Khodayar-Pardo [48]	Breast pump	qPCR	N/A	*Bifidobacterium* spp., *Enterococcus* spp., *Lactobacillus* spp., *Staphylococcus* spp., *Streptococcus* spp.	Stages of Lactation: Distinct changes in microbial composition and concentration between colostrum, transitional, and mature milk. High levels of microbes in colostrum predicted similarly high levels in later stages (*p* = 0.0001 for multiple genera). Gestational Age: Increasing microbial concentration with gestational age, although it was not statistically significant for preterm birth levels. Mode of Delivery: Vaginal delivery was associated with more frequent detection of *Bifidobacterium* spp. and *Streptococcus* spp. in colostrum.
Obermajer [54]	Manually	qPCR, cultures, 16S rRNA sequencing	Bacteria: 9.4 × 10^6^–1.1 × 10^9^ GE/mL	*Enterobacteriaceae*, *Clostridium cluster XIV*, *Bacteroides-Prevotella group*, *Bifidobacterium*, *Staphylococcus*, *Streptococcus*,	Bacterial Species *Staphylococcus epidermidis*: Found in 71.1% of colostrum samples. Presence of Bacteriocin Genes Salivaricin: Detected in a small number of samples (exact number not specified). Streptin: Found in a limited number of samples.
Cabrera-Rubio 2012 [31]	Manually	qPCR, 16S rRNA, Pyrosequencing	>1000 OTUs based on 97% sequence identity	*Weisella*, *Leuconostoc*, *Lactobacillales.*, *Staphylococcus*, *Streptococcus*, *Lactococcus*, *Bacilli*	Higher in colostrum compared to milk samples at 1 and 6 months. Lower diversity in colostrum from obese mothers. Correlations with Maternal BMI: *Lactobacillus* (r = 0.600, *p* = 0.026). *Staphylococcus* (r = 0.560, *p* = 0.038). Effect of Delivery Mode: Planned Cesarean: Reduced presence of *Leuconostocaceae* and increased presence of *Carnobacteriaceae*.
Collado [34]	Manually	qPCR	Normal BMI: Median 5.90 log gene copies/mL (IQR: 5.37–6.26). Overweight BMI: Median 6.18 log gene copies/mL (IQR: 6.00–6.35; *p* = 0.024).	*Weisella*, *Leuconostoc*, *Staphylococcus*, *Streptococcus και Lactococcus*, *Veillonella*, *Leptotrichia*, *Prevotella*,	IL-6: Positive correlation with the number of Staphylococcus in normal-weight mothers (r = 0.628, *p* = 0.039). Negative correlation with the number of *Akkermansia muciniphila* in overweight mothers (r = −0.738, *p* = 0.015). Lower *Bifidobacterium* colonies were associated with excessive weight gain during pregnancy (r = −0.629, *p* = 0.038).
Dubos [42]	N/A	PCR, 16S rDNA sequencing	Lactobacillus: 3.33 ± 0.55 logCFU/ml	*Lactobacillus*	Resistance to Gastric pH and Bile Salts: 28% of Lactobacillus strains were resistant to gastric pH (pH 2.0) and bile salts (0.4% Oxgall).
Solis [58]	Manually	Gene sequencing 16S rRNA, culture, RAPD-PCR.	Changes in Bacterial Count Over Time: Day 1: 5 log CFU/mL. Day 10: A decrease was observed, but it was not quantified. Day 90: 3.7 log CFU/mL. Bifidobacterium Levels in Breast Milk: Detected in 20% of the samples on Day 1 and in 60% of the samples on Days 10, 30, and 90. Concentrations ranged from 2.5 to 4.8 log CFU/mL.	*Streptococcus*, *Staphylococcus* *Lactobacillus*, *Bifidobacterium:*	Vertical Transmission: Identical genetic profiles of *Bifidobacterium* strains were found in both maternal milk and the corresponding infant feces, providing strong evidence for vertical bacterial transmission from mother to infant. Bacterial Persistence Over Time: Viable *Lactobacilli* and *Bifidobacterium* were consistently detected in breast milk, though less frequently than *Streptococcus* and *Staphylococcus*.
Martin [72]	N/A	16S rRNA	Bacteria: 2 × 10^4^–1 × 10^5^ CFU/mL	*Lactobacillus gasseri*, *Lactobacillus fermentum*, *Enterococcus faecium*	RAPD-PCR Findings: Identical RAPD Profiles in Mother–Infant Pair B: *Lactobacillus gasseri:* Seven isolates from maternal milk matched 16 from the areolar skin, 7 from stools, and 6 from oral samples. *Enterococcus faecium:* Two isolates from maternal milk matched three from the areolar skin, two from stools, and three from oral samples. Non-Matching RAPD Profiles: No matches were found between *lactobacilli* from breast skin or vaginal samples and those from other sources. Additional Observations: Total number of *lactobacilli* isolates: 300 from biological samples in mother–infant pairs. Detailed analysis was carried out on the following: Total of 78 isolates of *lactobacilli* in rod shape. Total of 100 isolates of *lactobacilli* in spherical shape.
Wyatt [70]	Manually	cultures	Bacterial cells: 10^3^–10^6^/mL	*Micrococci*, *Streptococcus*, *Lactobacillus*, *Enterobacteriaceae*	Conclusions: No significant differences were observed in the concentrations or types of bacteria between the following: Samples from the same breast. Samples at different stages of lactation. No trend of increase or decrease in the number of bacteria over time was detected, suggesting stable microbiome concentrations in the samples.

N/A; not applicable, PCR; polymerase chain reaction, qPCR; quantitative polymerase chain reaction, rRNA; ribosomal RNA, NMR ELISA; Nuclear Magnetic Resonance Enzyme-Linked Immunosorbent Assay, RAPD PCR; Random Amplified Polymorphic DNA Polymerase Chain Reaction, OTU; operational taxonomic unit, CFU; Colony-Forming Unit, GE; gene equivalents, HM; human milk, AUCg; area under the curve with respect to the ground, FDR; false discovery rate, STAI-S; State-Trait Anxiety Inventory scale, GAS; Geburts–Angst–Skala, HMO; human milk oligosaccharides, T1D; type 1 diabetes, w/o; without, CXM; Cefuroxime, CFX; Ceftriaxone, IAP; intrapartum antibiotic prophylaxis.

## Data Availability

Data are contained within the article.

## Supplementary Materials

The following supporting information can be downloaded at: https://www.mdpi.com/article/10.3390/children12101336/s1, PRISMA checklist; PROSPERO registered protocol.

## References

[1] Ballard O., Morrow A.L. (2013). Human Milk Composition. Pediatr. Clin. N. Am..

[2] Garofoli F., Civardi E., Pisoni C., Angelini M., Ghirardello S. (2023). Anti-Inflammatory and Anti-Allergic Properties of Colostrum from Mothers of Full-Term and Preterm Babies: The Importance of Maternal Lactation in the First Days. Nutrients.

[3] Castellote C., Casillas R., Ramírez-Santana C., Pérez-Cano F.J., Castell M., Moretones M.G., López-Sabater M.C., Franch Í (2011). Premature Delivery Influences the Immunological Composition of Colostrum and Transitional and Mature Human Milk. J. Nutr..

[4] Hassiotou F., Geddes D.T., Hartmann P.E. (2013). Cells in Human Milk. J. Hum. Lact..

[5] Hurley W.L., Theil P.K. (2011). Perspectives on Immunoglobulins in Colostrum and Milk. Nutrients.

[6] Bardanzellu F., Fanos V., Reali A. (2017). “Omics” in Human Colostrum and Mature Milk: Looking to Old Data with New Eyes. Nutrients.

[7] Duijts L., Jaddoe V.W.V., Hofman A., Moll H.A. (2010). Prolonged and Exclusive Breastfeeding Reduces the Risk of Infectious Diseases in Infancy. Pediatrics.

[8] Ladomenou F., Moschandreas J., Kafatos A., Tselentis Y., Galanakis E. (2010). Protective effect of exclusive breastfeeding against infections during infancy: A prospective study. Arch. Dis. Child..

[9] Meinzen-Derr J., Poindexter B., Wrage L., Morrow A.L., Stoll B., Donovan E.F. (2008). Role of human milk in extremely low birth weight infants’ risk of necrotizing enterocolitis or death. J. Perinatol..

[10] Munblit D., Verhasselt V. (2016). Allergy prevention by breastfeeding: Possible mechanisms and evidence from human cohorts. Curr. Opin. Allergy Clin. Immunol..

[11] Minniti F., Comberiati P., Munblit D., Piacentini G.L., Antoniazzi E., Zanoni L., Boner A.L., Peroni D.G. (2014). Breast-Milk Characteristics Protecting Against Allergy. Endocr. Metab. Immune Disord. Drug Targets.

[12] Gillman M.W. (2001). Risk of Overweight Among Adolescents Who Were Breastfed as Infants. JAMA.

[13] Kelishadi R., Farajian S. (2014). The protective effects of breastfeeding on chronic non-communicable diseases in adulthood: A review of evidence. Adv. Biomed. Res..

[14] Anne R.P., Kumar J., Kumar P., Meena J. (2023). Effect of oropharyngeal colostrum therapy on neonatal sepsis in preterm neonates: A systematic review and meta-analysis. J. Pediatr. Gastroenterol. Nutr..

[15] Kumar J., Meena J., Ranjan A., Kumar P. (2023). Oropharyngeal application of colostrum or mother’s own milk in preterm infants: A systematic review and meta-analysis. Nutr. Rev..

[16] Fu Z.Y., Huang C., Lei L., Chen L.C., Wei L.J., Zhou J., Martins C.C. (2023). The effect of oropharyngeal colostrum administration on the clinical outcomes of premature infants: A meta-analysis. Int. J. Nurs. Stud..

[17] Togo A., Grine G., Khelaifia S., des Robert C., Brevaut V., Caputo A., Baptiste E., Bonnet M., Levasseur A., Drancourt M. (2019). Culture of Methanogenic Archaea from Human Colostrum and Milk. Sci. Rep..

[18] Pannaraj P.S., Li F., Cerini C., Bender J.M., Yang S., Rollie A., Adisetiyo H., Zabih S., Lincez P.J., Bittinger K. (2017). Association Between Breast Milk Bacterial Communities and Establishment and Development of the Infant Gut Microbiome. JAMA Pediatr..

[19] Martín V., Maldonado-Barragán A., Moles L., Rodriguez-Baños M., Campo Rdel Fernández L., Fernández L., Jiménez E., Fernández M., Rodríguez J.M., Delgado S. (2012). Sharing of Bacterial Strains Between Breast Milk and Infant Feces. J. Hum. Lact..

[20] Zimmermann P., Curtis N. (2018). The influence of the intestinal microbiome on vaccine responses. Vaccine.

[21] Stewart C.J., Ajami N.J., O’Brien J.L., Hutchinson D.S., Smith D.P., Wong M.C., Ross M.C., Lloyd R.E., Doddapaneni H., Metcalf G.A. (2018). Temporal development of the gut microbiome in early childhood from the TEDDY study. Nature.

[22] Arrieta M.C., Stiemsma L.T., Amenyogbe N., Brown E.M., Finlay B. (2014). The Intestinal Microbiome in Early Life: Health and Disease. Front. Immunol..

[23] Knights D., Lassen K.G., Xavier R.J. (2013). Advances in inflammatory bowel disease pathogenesis: Linking host genetics and the microbiome. Gut.

[24] Ridaura V.K., Faith J.J., Rey F.E., Cheng J., Duncan A.E., Kau A.L., Griffin N.W., Lombard V., Henrissat B., Bain J. (2013). Gut Microbiota from Twins Discordant for Obesity Modulate Metabolism in Mice. Science.

[25] Kostic A.D., Gevers D., Siljander H., Vatanen T., Hyötyläinen T., Hämäläinen A.M., Peet A., Tillmann V., Salo H., Virtanen S.M. (2015). The Dynamics of the Human Infant Gut Microbiome in Development and in Progression toward Type 1 Diabetes. Cell Host Microbe.

[26] Harris V., Ali A., Fuentes S., Korpela K., Kazi M., Tate J., Arakawa Y., Axelin A., Bode L., Browne H. (2017). Rotavirus vaccine response correlates with the infant gut microbiota composition in Pakistan. Gut Microbes.

[27] Grainger M., Haddaway N. (2025). Evidence Synthesis Hackathon. PRISMA2020. https://www.eshackathon.org/software/PRISMA2020.html.

[28] Urashima T., Ajisaka K., Ujihara T., Nakazaki E. (2025). Recent advances in the science of human milk oligosaccharides. BBA Adv..

[29] Boix-Amorós A., Collado M.C., Mira A. (2016). Relationship between Milk Microbiota, Bacterial Load, Macronutrients, and Human Cells during Lactation. Front. Microbiol..

[30] Boix-Amorós A., Martinez-Costa C., Querol A., Collado M.C., Mira A. (2017). Multiple Approaches Detect the Presence of Fungi in Human Breastmilk Samples from Healthy Mothers. Sci. Rep..

[31] Cabrera-Rubio R., Collado M.C., Laitinen K., Salminen S., Isolauri E., Mira A. (2012). The human milk microbiome changes over lactation and is shaped by maternal weight and mode of delivery. Am. J. Clin. Nutr..

[32] Cabrera-Rubio R., Kunz C., Rudloff S., García-Mantrana I., Crehuá-Gaudiza E., Martínez-Costa C. (2019). Association of Maternal Secretor Status and Human Milk Oligosaccharides With Milk Microbiota. J. Pediatr. Gastroenterol. Nutr..

[33] Chen P.W., Lin Y.L., Huang M.S. (2018). Profiles of commensal and opportunistic bacteria in human milk from healthy donors in Taiwan. J. Food Drug Anal..

[34] Collado M.C., Laitinen K., Salminen S., Isolauri E. (2012). Maternal weight and excessive weight gain during pregnancy modify the immunomodulatory potential of breast milk. Pediatr. Res..

[35] Corona-Cervantes K., García-González I., Villalobos-Flores L.E., Hernández-Quiroz F., Piña-Escobedo A., Hoyo-Vadillo C., Rangel-Calvillo M.N., García-Mena J. (2020). Human milk microbiota associated with early colonization of the neonatal gut in Mexican newborns. PeerJ.

[36] Damaceno Q.S., Souza J.P., Nicoli J.R., Paula R.L., Assis G.B., Figueiredo H.C., Azevedo V., Martins F.S. (2017). Evaluation of Potential Probiotics Isolated from Human Milk and Colostrum. Probiotics Antimicrob. Proteins.

[37] Davé V., Street K., Francis S., Bradman A., Riley L., Eskenazi B. (2016). Bacterial microbiome of breast milk and child saliva from low-income Mexican-American women and children. Pediatr. Res..

[38] Deflorin N., Ehlert U., Amiel Castro R.T. (2025). Associations of Maternal Salivary Cortisol and Psychological Symptoms With Human Milk’s Microbiome Composition. Biopsychosoc. Sci. Med..

[39] Dewanto N.E.F., Firmansyah A., Sungkar A., Dharmasetiawani N., Sastroasmoro S., Kresno S.B., Hadi U. (2017). The effect of *Bifidobacterium animalis lactis* HNO19 supplementation among pregnant and lactating women on interleukin-8 level in breast milk and infant’s gut mucosal integrity. Med. J. Indones..

[40] Drago L., Toscano M., De Grandi R., Grossi E., Padovani E.M., Peroni D.G. (2016). Microbiota network and mathematic microbe mutualism in colostrum and mature milk collected in two different geographic areas: Italy versus Burundi. ISME J..

[41] Du Y., Qiu Q., Cheng J., Huang Z., Xie R., Wang L., Li X., Zhang Y., Chen H., Liu J. (2022). Comparative study on the microbiota of colostrum and nipple skin from lactating mothers separated from their newborn at birth in China. Front. Microbiol..

[42] Dubos C., Vega N., Carvallo C., Navarrete P., Cerda C., Brunser O., Gotteland M. (2011). Identification of Lactobacillus spp. in colostrum from Chilean mothers. Arch. Latinoam. Nutr..

[43] Fernández-Tuñas Mdel C., Pérez-Muñuzuri A., Trastoy-Pena R., Pérez del Molino M.L., Couce M.L. (2023). Effects of Maternal Stress on Breast Milk Production and the Microbiota of Very Premature Infants. Nutrients.

[44] Gámez-Valdez J.S., García-Mazcorro J.F., Montoya-Rincón A.H., Rodríguez-Reyes D.L., Jiménez-Blanco G., Rodríguez M.T.A., López R., Torres E., Hernández C., Sánchez P. (2021). Differential analysis of the bacterial community in colostrum samples from women with gestational diabetes mellitus and obesity. Sci. Rep..

[45] Ge H., Zhu W., Zhang J., Wang Z., Shi H., Sun J., Li Y., Chen X., Liu Q., Huang M. (2024). Human milk microbiota and oligosaccharides in colostrum and mature milk: Comparison and correlation. Front. Nutr..

[46] Ji C., Zhang G., Xu S., Xiang Q., Huang M., Zhao M., Li Y., Chen H., Wang L., Liu J. (2022). Antibiotic treatments to mothers during the perinatal period leaving hidden trouble on infants. Eur. J. Pediatr..

[47] Karampatsas K., Faal A., Jaiteh M., Garcia-Perez I., Aller S., Shaw A.G., Kampmann B., Prentice A., Moore S.E., Lloyd-Price J. (2022). Gastrointestinal, vaginal, nasopharyngeal, and breast milk microbiota profiles and breast milk metabolomic changes in Gambian infants over the first two months of lactation: A prospective cohort study. Medicine.

[48] Khodayar-Pardo P., Mira-Pascual L., Collado M.C., Martínez-Costa C. (2014). Impact of lactation stage, gestational age and mode of delivery on breast milk microbiota. J. Perinatol..

[49] Li Y., Ren L., Wang Y., Li J., Zhou Q., Peng C., Zhang H., Liu X., Chen J., Huang Y. (2022). The Effect of Breast Milk Microbiota on the Composition of Infant Gut Microbiota: A Cohort Study. Nutrients.

[50] Liu B., Zhao J., Liu Y., Qiao W., Jiang T., Chen L. (2022). Diversity and temporal dynamics of breast milk microbiome and its influencing factors in Chinese women during the first 6 months postpartum. Front. Microbiol..

[51] Mastromarino P., Capobianco D., Miccheli A., Praticò G., Campagna G., Laforgia N., Marcellini S., Salimei E., Menghini P., Mattarelli P. (2015). Administration of a multistrain probiotic product (VSL#3) to women in the perinatal period differentially affects breast milk beneficial microbiota in relation to mode of delivery. Pharmacol. Res..

[52] Moles L., Manzano S., Fernández L., Montilla A., Corzo N., Ares S., Rueda R., Maldonado A., Rodríguez M., Martín V. (2015). Bacteriological, biochemical, and immunological properties of colostrum and mature milk from mothers of extremely preterm infants. J. Pediatr. Gastroenterol. Nutr..

[53] Nikolopoulou G., Tsironi T., Halvatsiotis P., Petropoulou E., Genaris N., Vougiouklaki D., Koutelidakis A., Papadimitriou A., Vasileiou A., Antoniou C. (2021). Analysis of the major probiotics in healthy women’s breast milk by realtime PCR: Factors affecting the presence of those bacteria. Appl. Sci..

[54] Obermajer T., Lipoglavšek L., Tompa G., Treven P., Lorbeg P.M., Matijašić B.B., Rupnik M., Novak F., Klančar U., Štrukelj B. (2014). Colostrum of healthy Slovenian mothers: Microbiota composition and bacteriocin gene prevalence. PLoS ONE.

[55] Qi C., Zhou J., Tu H., Tu R., Chang H., Chen J., Li X., Wang Y., Zhang L., Liu M. (2022). Lactation-dependent vertical transmission of natural probiotics from the mother to the infant gut through breast milk. Food Funct..

[56] Sakwinska O., Moine D., Delley M., Combremont S., Rezzonico E., Descombes P., Vinyes-Pares G., Zhang Y., Wang P., Thakkar S.K. (2016). Microbiota in Breast Milk of Chinese Lactating Mothers. PLoS ONE.

[57] Singh P., Al Mohannadi N., Murugesan S., Almarzooqi F., Kabeer B.S.A., Marr A.K., Kino T., Brummaier T., Terranegra A., McGready R. (2023). Unveiling the dynamics of the breast milk microbiome: Impact of lactation stage and gestational age. J. Transl. Med..

[58] Solís G., de Los Reyes-Gavilan C.G., Fernández N., Margolles A., Gueimonde M. (2010). Establishment and development of lactic acid bacteria and bifidobacteria microbiota in breast-milk and the infant gut. Anaerobe.

[59] Sun W., Tao L., Qian C., Xue P.P., Du S.S., Tao Y.N. (2024). Human milk oligosaccharides: Bridging the gap in intestinal microbiota between mothers and infants. Front. Cell Infect. Microbiol..

[60] Tang M., Xu C., Chen K., Yan Q., Mao W., Liu W., Ritz B. (2019). Hexachlorocyclohexane exposure alters the microbiome of colostrum in Chinese breastfeeding mothers. Environ. Pollut..

[61] Tao Y.N., Tong X.K., Qian C., Wan H., Zuo J.P. (2020). Microbial quantitation of colostrum from healthy breastfeeding women and milk from mastitis patients. Ann. Palliat. Med..

[62] Tapia-González A., Vélez-Ixta J.M., Bueno-Hernández N., Piña-Escobedo A., Briones-Garduño J.C., de la Rosa-Ruiz L., Aguayo-Guerrero J., Mendoza-Martínez V.M., Snowball-del-Pilar L., Escobedo G. (2023). Maternal Consumption of Non-Nutritive Sweeteners during Pregnancy Is Associated with Alterations in the Colostrum Microbiota. Nutrients.

[63] Toscano M., De Grandi R., Peroni D.G., Grossi E., Facchin V., Comberiati P., Drago L. (2017). Impact of delivery mode on the colostrum microbiota composition. BMC Microbiol..

[64] Tuominen H., Rautava S., Collado M.C., Syrjänen S., Rautava J. (2018). HPV infection and bacterial microbiota in breast milk and infant oral mucosa. PLoS ONE.

[65] Wan Y., Jiang J., Lu M., Tong W., Zhou R., Li J., Yuan J., Wang F., Li D. (2020). Human milk microbiota development during lactation and its relation to maternal geographic location and gestational hypertensive status. Gut Microbes.

[66] Wang Y., Wang J., Yu D., Zou J., Zhang C., Yan H., Ye X., Chen Y. (2022). Microbial community structure of colostrum in women with antibiotic exposure immediately after delivery. Breastfeed. Med..

[67] Wang K., Xia X., Sun L., Wang H., Li Q., Yang Z., Ren J. (2023). Microbial diversity and correlation between breast milk and the infant gut. Foods.

[68] Williams J.E., Carrothers J.M., Lackey K.A., Beatty N.F., York M.A., Brooker S.L., Peterson H.K., Eppensteiner E., Serao M.C., Kellermayer R. (2017). Human milk microbial community structure is relatively stable and related to variations in macronutrient and micronutrient intakes in healthy lactating women. J. Nutr..

[69] Williams J.E., Carrothers J.M., Lackey K.A., Beatty N.F., Brooker S.L., Peterson H.K., Eppensteiner E., Serao M.C., Kellermayer R., Bode L. (2019). Strong multivariate relations exist among milk, oral, and fecal microbiomes in mother-infant dyads during the first six months postpartum. J. Nutr..

[70] Wyatt R.G., Mata L.J. (1969). Bacteria in colostrum and milk of Guatemalan Indian women. J. Trop. Pediatr..

[71] Xie W., Zhang H., Ni Y., Peng Y. (2022). Contrasting Diversity and Composition of Human Colostrum Microbiota in a Maternal Cohort With Different Ethnic Origins but Shared Physical Geography (Island Scale). Front. Microbiol..

[72] Martín R., Langa S., Reviriego C., Jiménez E., Marín M.L., Xaus J., Fernández L., Rodríguez J.M. (2003). Human milk is a source of lactic acid bacteria for the infant gut. J. Pediatr..

[73] Aakko J., Kumar H., Rautava S., Wise A., Autran C., Bode L., Isolauri E., Salminen S. (2017). Human Milk Oligosaccharide Categories Define the Microbiota Composition in Human Colostrum. Benef. Microbes.

[74] Ramsay D.T., Kent J.C., Owens R.A., Hartmann P.E. (2004). Ultrasound Imaging of Milk Ejection in the Breast of Lactating Women. Pediatrics.

[75] Cortez R.V., Fernandes A., Sparvoli L.G., Padilha M., Feferbaum R., Neto C.M., Taddei C.R. (2021). Impact of oropharyngeal administration of colostrum in preterm newborns’ oral microbiome. Nutrients.

[76] Xu R., McLoughlin G., Nicol M., Geddes D., Stinson L. (2024). Residents or tourists: Is the lactating mammary gland colonized by residential microbiota?. Microorganisms.

[77] Perez P.F., Doré J., Leclerc M., Levenez F., Benyacoub J., Serrant P., Segura-Roggero I., Schiffrin E.J., Donnet-Hughes A. (2007). Bacterial imprinting of the neonatal immune system: Lessons from maternal cells?. Pediatrics.

[78] Fernandes A., Jobby R. (2022). Bacteriocins from lactic acid bacteria and their potential clinical applications. Appl. Biochem. Biotechnol..

[79] Arqués J.L., Rodríguez E., Langa S., Landete J.M., Medina M. (2015). Antimicrobial Activity of Lactic Acid Bacteria in Dairy Products and Gut: Effect on Pathogens. BioMed Res. Int..

[80] Reis N.A., Saraiva Ma F., Duarte Ea A., de Carvalho E.A., Vieira B.B., Evangelista-Barreto N.S. (2016). Probiotic properties of lactic acid bacteria isolated from human milk. J. Appl. Microbiol..

[81] Yu H.S., Lee N.K., Choi A.J., Choe J.S., Bae C.H., Paik H.D. (2019). Anti-Inflammatory Potential of Probiotic Strain Weissella cibaria JW15 Isolated from Kimchi through Regulation of NF-κB and MAPKs Pathways in LPS-Induced RAW 264.7 Cells. J. Microbiol. Biotechnol..

[82] Suárez-Martínez C., Santaella-Pascual M., Yagüe-Guirao G., Martínez-Graciá C. (2023). Infant gut microbiota colonization: Influence of prenatal and postnatal factors, focusing on diet. Front. Microbiol..

[83] Kalliomäki M., Kirjavainen P., Eerola E., Kero P., Salminen S., Isolauri E. (2001). Distinct patterns of neonatal gut microflora in infants in whom atopy was and was not developing. J. Allergy Clin. Immunol..

[84] Odiase E., Frank D.N., Young B.E., Robertson C.E., Kofonow J.M., Davis K.N., Schuster M., Li J., Nicholson J.K., Smith K. (2023). The gut microbiota differ in exclusively breastfed and formula-fed United States infants and are associated with growth status. J. Nutr..

[85] Hayen S.M., den Hartog Jager C.F., Knulst A.C., Knol E.F., Garssen J., Willemsen L.E.M., van Esch B.C.A.M., Pasmans S.G.M.A., Knipping K., Savelkoul H.F.J. (2018). Non-digestible oligosaccharides can suppress basophil degranulation in whole blood of peanut-allergic patients. Front. Immunol..

[86] Heikkila M.P., Saris P.E.J. (2003). Inhibition of Staphylococcus aureus by the commensal bacteria of human milk. J. Appl. Microbiol..

