# Supplementary material for: Factors Influencing the Colostrum’s Microbiota: A Systematic Review of the Literature
Source: Children (Basel). 2025 Oct 4;12(10):1336. doi: 10.3390/children12101336 (PMC12562569; doi:10.3390/children12101336)
Supplement: Supplementary file 1 [file children-12-01336-s001.zip › PROSPERO.pdf]

NIHR | National Institute for Health and Care Research

PROSPERO
International prospective register of systematic reviews

Factors influencing the colostrum's microbiota: A systematic review of the literature

Eleni Karapati, Aimilia Tzani

To enable PROSPERO to focus on COVID-19 submissions, this registration record has undergone basic automated checks for eligibility and is published exactly as submitted. PROSPERO has never provided peer review, and usual checking by the PROSPERO team does not endorse content. Therefore, automatically published records should be treated as any other PROSPERO registration. Further detail is provided here.

Citation
Eleni Karapati, Aimilia Tzani. Factors influencing the colostrum's microbiota: A systematic review of the literature. PROSPERO 2024 Available from https://www.crd.york.ac.uk/PROSPERO/view/CRD42025644017

REVIEW TITLE AND BASIC DETAILS

Review title
Factors influencing the colostrum's microbiota: A systematic review of the literature

Review objectives
This systematic review aims to summarize studies on colostrum microbiota in humans, exploring both intrinsic and extrinsic factors that may shape it.

SEARCHING AND SCREENING

Searches
An electronic review of the current literature to identify relevant articles will be conducted, utilizing the Scopus and PubMed databases as well as manual research. Articles were screened for eligibility based on predefined inclusion and exclusion criteria. Only original

studies investigating the overall microbial composition of human colostrum, defined as milk collected within the first five days postpartum, will be included. Only studies in English will be included, and no geographical or time limitations will be implemented.
Studies are excluded if they (i)analyzed pasteurized milk; (ii) did not clearly specify the type of milk analyzed; or (iii) involved milk collected after five days postpartum. Additionally, reference lists of selected articles will be reviewed to identify other potentially relevant studies.
The following data will be extracted from each included study: publication year, country of research, participant characteristics (e.g., maternal age, prior antibiotic use, probiotic supplementation, gestational age, mode of delivery, feeding method), milk sample size, timing and method of milk collection, microbiota analysis techniques, and key findings related to microbial diversity, abundance, and associations with influencing factors.

Study design

All observational studies (cohort studies, cross-sectional studies, case-control studies, case series, case
reports) and clinical trials referring to colostrum's microbiota will be included.
Review articles, systematic reviews and meta-analyses, as well as conference proceedings will be excluded.

ELIGIBILITY CRITERIA

Condition or domain being studied

Microflora of Human colostrum, the initial milk form- produced postpartum.

Population

original studies investigating the overall microbial composition of human colostrum, defined as milk collected within the first five days postpartum

Intervention(s) or exposure(s)

microbiota of colostrum (

Comparator(s) or control(s)

none

OUTCOMES TO BE ANALYSED

Main outcomes

1.colostrum microbiota identification
2.factors that influence colostrum colonization

Additional outcomes

none

DATA COLLECTION PROCESS

Data extraction (selection and coding)

All the identified articles will be added in EndNote Program; duplicates will be removed. Retrieved studies will
be initially screened for eligibility based on title and abstract; irrelevant studies will be excluded. Two
independent researchers will evaluate the full text of the remaining articles to identify those meeting the
inclusion criteria for the review; possible discrepancies will be resolved with the aid of a third investigator

Risk of bias (quality) assessment

The quality of eligible studies will be evaluated using: the Strengthening the Reporting of Observational
Studies in Epidemiology (STROBE) for cohort, case-control and cross-sectional studies, Cochrane risk-of
bias (RoB 2) for clinical trials and the JBI Critical Appraisal Checklist for Case Reports.

PLANNED DATA SYNTHESIS

Strategy for data synthesis

Retrieved data will be extracted into a Microsoft Excel template. Meta-analysis will be conducted only if
extracted data from a subset of studies are sufficiently homogenous in terms of patient characteristics,
interventions and outcome.

Analysis of subgroups or subsets

none

REVIEW AFFILIATION, FUNDING AND PEER REVIEW

Review team members

- Dr Eleni Karapati, Aretaieion Hospital
- Aimilia Tzani, Division of Pediatrics, Geneva University Hospital (HUG)

Review affiliation

Aretaieion Hospital

Funding source

no funding

Named contact
Eleni Karapati. Papaflessa 3, Melissia, Athens, Greece
helenak5@hotmail.com

TIMELINE OF THE REVIEW

Review timeline
Start date: 01 December 2024. End date: 26 February 2025

Date of first submission to PROSPERO
28 January 2025

Date of registration in PROSPERO
08 February 2025

CURRENT REVIEW STAGE

Publication of review results
The intention is not to publish the review once completed.

Stage of the review at this submission

Review stage	Started	Completed
Pilot work	✓	
Formal searching/study identification	✓	
Screening search results against inclusion criteria		
Data extraction or receipt of IP		
Risk of bias/quality assessment		
Data synthesis		

Review status
The review is currently planned or ongoing.

ADDITIONAL INFORMATION

PROSPERO version history

- Version 1.1 published on 08 Feb 2025
- Version 1.0 published on 08 Feb 2025

Review conflict of interest
None known

Country
Greece

Disclaimer
The content of this record displays the information provided by the review team. PROSPERO does not peer review registration records or endorse their content.

PROSPERO accepts and posts the information provided in good faith; responsibility for record content rests with the review team. The owner of this record has affirmed that the information provided is truthful and that they understand that deliberate provision of inaccurate information may be construed as scientific misconduct.

PROSPERO does not accept any liability for the content provided in this record or for its use. Readers use the information provided in this record at their own risk.

Any enquiries about the record should be referred to the named review contact
